# Chromatin profiling reveals TFAP4 as a critical transcriptional regulator of bovine satellite cell differentiation

**DOI:** 10.1186/s12864-024-10189-2

**Published:** 2024-03-12

**Authors:** Pengcheng Lyu, Honglin Jiang

**Affiliations:** School of Animal Sciences, Virginia Polytechnic Institute and State University, Blacksburg, VA 24061 USA

**Keywords:** Bovine, Chromatin, Enhancers, Histone modification, Muscle, Transcription factors

## Abstract

**Background:**

Satellite cells are myogenic precursor cells in adult skeletal muscle and play a crucial role in skeletal muscle regeneration, maintenance, and growth. Like embryonic myoblasts, satellite cells have the ability to proliferate, differentiate, and fuse to form multinucleated myofibers. In this study, we aimed to identify additional transcription factors that control gene expression during bovine satellite cell proliferation and differentiation.

**Results:**

Using chromatin immunoprecipitation followed by sequencing, we identified 56,973 and 54,470 genomic regions marked with both the histone modifications H3K4me1 and H3K27ac, which were considered active enhancers, and 50,956 and 59,174 genomic regions marked with H3K27me3, which were considered repressed enhancers, in proliferating and differentiating bovine satellite cells, respectively. In addition, we identified 1,216 and 1,171 super-enhancers in proliferating and differentiating bovine satellite cells, respectively. Analyzing these enhancers showed that in proliferating bovine satellite cells, active enhancers were associated with genes stimulating cell proliferation or inhibiting myoblast differentiation whereas repressed enhancers were associated with genes essential for myoblast differentiation, and that in differentiating satellite cells, active enhancers were associated with genes essential for myoblast differentiation or muscle contraction whereas repressed enhancers were associated with genes stimulating cell proliferation or inhibiting myoblast differentiation. Active enhancers in proliferating bovine satellite cells were enriched with binding sites for many transcription factors such as MYF5 and the AP-1 family transcription factors; active enhancers in differentiating bovine satellite cells were enriched with binding sites for many transcription factors such as MYOG and TFAP4; and repressed enhancers in both proliferating and differentiating bovine satellite cells were enriched with binding sites for NF-kB, ZEB-1, and several other transcription factors. The role of TFAP4 in satellite cell or myoblast differentiation was previously unknown, and through gene knockdown and overexpression, we experimentally validated a critical role for TFAP4 in the differentiation and fusion of bovine satellite cells into myofibers.

**Conclusions:**

Satellite cell proliferation and differentiation are controlled by many transcription factors such as AP-1, TFAP4, NF-kB, and ZEB-1 whose roles in these processes were previously unknown in addition to those transcription factors such as MYF5 and MYOG whose roles in these processes are widely known.

**Supplementary Information:**

The online version contains supplementary material available at 10.1186/s12864-024-10189-2.

## Background

Satellite cells are myogenic precursor cells residing in adult skeletal muscle [1]. In response to stimuli such as injury, the normally dormant satellite cells are activated, proliferate, differentiate, and fuse with each other to form new multinucleated myofibers or with existing myofibers to repair damaged muscle [2, 3]. Besides playing an essential role in skeletal muscle regeneration, satellite cells contribute to the maintenance and growth of skeletal muscle in postnatal animals [3].

Little is known about the transcription factors that control gene expression during satellite cell proliferation. Gene expression during satellite cell differentiation in postnatal animals is believed to be controlled by the same transcription factors that control myoblast differentiation in the embryos [2, 3]. These transcription factors include the myogenic regulatory factor myogenin (MYOG or MyoG) [4] and the myocyte enhancer factor 2 (MEF2) family of transcription factors [5]. An earlier ChIP-on-chip study identified nearly 200 genes directly targeted by MYOG or MEF2s in differentiating C2C12 myoblasts [6]. However, based on RNA sequencing, much more than 200 genes are upregulated during satellite cell differentiation and fusion into myofibers [7, 8]. The large difference between the number of genes targeted by MYOG and MEF2s and that of genes upregulated during satellite cell differentiation suggests that gene expression during satellite cell differentiation is regulated by many transcription factors in addition to MYOG and MEF2s.

Transcription factors regulate gene transcription by binding to short DNA sequences called transcription factor binding sites or motifs in regulatory DNA regions called enhancers and by recruiting coactivators that modify histones at the enhancers and hence chromatin structure and accessibility [9, 10]. Consequently, enhancers are often associated with histone modifications [11]. Specifically, enhancers that are actively participating in gene transcription, i.e., active enhancers, are associated with acetylation of lysine 27 of histone 3 protein (H3K27ac) [12]; active enhancers and primed enhancers (i.e., enhancers primed to be active) are associated with mono-methylation of lysine 4 of histone 3 (H3K4me1) [13]; and enhancers whose activity is repressed, i.e., repressed enhancers, are often associated with tri-methylation of lysine 27 of histone 3 (H3K27me3) [14]. Therefore, enhancers, transcription factor binding sites located in enhancers, and transcription factors binding to enhancers can be identified by mapping the genomic regions marked with specific histone modifications.

In this study, we performed a genome-wide profiling of chromatin associated with H3K27ac, H3K4me1, and H3K27me3 in proliferating and differentiating bovine satellite cells that were undergoing proliferation and differentiation, respectively. Bovine satellite cells were used in this study because they can be easily obtained in large amounts and because data from studying bovine satellite cells have direct implications for how to improve skeletal muscle growth and regeneration in cattle, which are important meat-producing animals. Our analyses of these chromatin modification data indicated that gene transcription in proliferating and differentiating bovine satellite cells is each controlled by a large set of transcription factors and that gene expression during bovine satellite cell differentiation is controlled by many transcription factors besides the widely known MYOG and MEF2s. We also experimentally demonstrated that the transcription factor TFAP4, also known as AP4 or AP-4, whose function in satellite cell or myoblast differentiation was previously unknown, is critical for bovine satellite cell differentiation.

## Methods

### Reagents, materials, and equipment

All reagents, materials, and equipment used in this study were purchased from ThermoFisher Scientific (Waltham, MA, USA) unless specified otherwise.

### Isolation and culture of bovine satellite cells

Skeletal muscles (the extensor carpi radialis muscles) were collected from adult Angus-crossbred steers at slaughter. Satellite cells were isolated through enzymatic digestion and differential centrifugation as previously described [15]. Satellite cells were cultured to proliferate in growth medium comprising Dulbecco’s Modified Eagle Medium (DMEM), 10% fetal bovine serum (FBS) (R&D Systems, Minneapolis, MN, USA), 2 mM L-glutamine, and 1% of 100× antibiotic-antimycotic (ABAM). To differentiate and fuse into myofibers, bovine satellite cells were cultured to approximately 80% confluency in growth medium and then in differentiation medium consisting of DMEM, 2% horse serum (R&D Systems), 2 mM L-glutamine, and 1% ABAM for two days. Based on a previous study [16], nearly 40% of bovine satellite cells would differentiate and fuse with each other to form myofibers containing 2 or more nuclei after 48-hour culture in differentiation medium. All animal-related procedures were approved by the Virginia Tech Institutional Animal Care and Use Committee under the IACUC number 20–169.

### Chromatin immunoprecipitation (ChIP)

Chromatin for ChIP was prepared as described previously [7]. Briefly, bovine satellite cells cultured in growth medium at approximately 80% confluency or in differentiation medium for 2 days were scraped in phosphate buffered saline (PBS) and then incubated with 1% formaldehyde in PBS for 10 min at room temperature to cross-link DNA-protein interaction. Cross-linking was quenched by adding 125 mM glycine. Cell nuclei were isolated by incubating cells with a hypotonic buffer (10 mM HEPES pH 7.5, 2 mM MgCl_2_, and 25 mM KCl) supplemented with a protease inhibitor cocktail on ice for 30 min followed by Dounce homogenization. Cell nuclei were sheared in ice-cold ChIP buffer from Diagenode (Denville, NJ, USA) to generate chromatin fragments of 200–1000 bp using a Model 100 sonic dismembrator.

ChIP was performed as described previously [7]. To describe the procedure again, chromatin fragments prepared from approximately 1 million cells were incubated with 2 µg of antibody against H3K27ac, H3K4me1, or H3K27me3 in a total volume of 300 µl containing bovine serum albumin (BSA), protease inhibitor cocktail, ChIP buffer iC1, and protein A-coated magnetic beads in addition to chromatin and antibody at 4 °C overnight with gentle rocking. Antibodies for H3K27ac, H3K4me1, and H3K27me3 were purchased from Diagenode (Catalog numbers C15210016, C15410037, and C15200181, respectively) and were used at approximately 1:150 dilution. All reagents for ChIP assays were from the iDeal ChIP-seq Kit for Histones from Diagenode (Catalog number C01010051). The remaining steps of ChIP, including washing, elution, cross-linking reversal, and DNA purification were performed using reagents from Diagenode, essentially according to the manufacturer’s instructions. Input DNA was prepared the same way as ChIP DNA except that the immunoprecipitation step was omitted.

### ChIP‑seq library construction and sequencing

ChIP-seq libraries were prepared from 1 to 5 ng of ChIP or Input DNA using the NEBNext Ultra II DNA Library Prep Kit for Illumina (New England BioLabs, Ipswich, MA, USA). Steps including end prep, adaptor ligation, cleanup of adaptor-ligated DNA without size selection, PCR enrichment of adaptor-ligated DNA (8 cycles), and cleanup of PCR reactions were performed essentially according to the manufacturer’s instructions. A total of 12 ChIP-seq libraries were prepared for three histone marks (i.e., H3K27ac, H3K4me1, and H3K27me3), two conditions (i.e., proliferation and differentiation), and two biological replicates (i.e., cells from two cattle). A total of 4 Input-seq libraries were made for two conditions and two biological replicates. DNA concentration of each library was determined with a Qubit 4 Fluorometer and quantitative PCR. DNA size distribution of each library was determined by running a sample on a TapeStation system (Agilent Technologies, Santa Clara, CA, USA). ChIP-seq libraries were pooled and pair-end sequenced on an Illumina NovaSeq at Novogene (Sacramento, CA, USA).

### ChIP‑seq data analyses

ChIP-seq data were analyzed as described previously with some modifications [7]. Sequences from each ChIP-seq library were trimmed to remove the adapters using Trimmomatic (0.39) [17]. The trimmed sequences were aligned to the reference bovine genome (assembly BosTau 9) using Hisat2 (2.2.0) [18]. The uniquely aligned sequences were sorted and merged using SAMtools (1.9) [19]. Peak calling was made using MACS3 (3.0.0a5) [20], where a ChIP-seq library was compared to an Input library constructed from the same chromatin sample. Quality of enrichment in each ChIP-seq library was assessed using Phantompeakqualtools (2.0) [21]. Overlapping ChIP-seq peaks were identified using bedtools intersect (2.29.2) [22]. ChIP-seq peaks were visualized in the IGV browser (2.8.2) [23] and annotated using ChIPseeker (1.22.1) [24]. Super enhancers were identified using ROSE (1.3.1) [25]. GO analysis was performed using the R package clusterProfiler (4.8.3) [26]. Motif enrichment analysis was performed using HOMER (4.11.1) [27]. Specific transcription factor binding sites were identified using the FIMO software (5.1.1) [22]. All analyses were performed at default parameters unless specified otherwise.

### Gene overexpression and knockdown

Overexpression of TFAP4 in bovine satellite cells was achieved by transfecting cells in 12-well plates with 0.5 µg of a TFAP4 expression plasmid (Catalog # TCM1304 from Transomic Technologies, Huntsville, AL, USA) using the transfection reagent Lipofectamine 3000 (ThermoFisher Scientific). Control cells were transfected with an EGFP expression plasmid (pcDNA3-EGFP from Addgene, Watertown, MA, USA). Knockdown of TFAP4 in bovine satellite cells was achieved by transfecting cells with 10 nM of a bovine TFAP4 mRNA-specific siRNA duplex using the transfection reagent Lipofectamine RNAiMAX (ThermoFisher Scientific). The sense and antisense sequences of the TFAP4 siRNA duplex were CACUCAGAAGGUGCCCUCUUUGCAA and UUGCAAAGAGGGCACCUUCUGAGUG, respectively. Control cells were transfected with a universal negative control siRNA duplex (MISSION siRNA Universal Negative Control #1, MilliporeSigma, Burlington, MA, USA). Cells were transfected in growth medium. Following overnight culture, cells were induced to differentiate into myofibers by changing growth medium to differentiation medium. Cells on day 4 of differentiation were collected for confirmation of TFAP4 overexpression or knockdown and other analyses described below.

### RNA extraction and reverse transcription-quantitative PCR

Total RNA was extracted using the Direct-zol RNA Miniprep Kit (Zymo Research, Irvine, CA, USA), essentially according to the manufacturer’s instruction. Reverse transcription was performed using the ImProm-II reverse transcriptase and random primers (Promega, Madison, WI, USA), according to the manufacturer’s instructions. Quantitative PCR (qPCR) was performed using the SYBR Green chemistry as described previously [7]. The relative expression levels of mRNAs were calculated using the 2^−ΔΔCt^ method [28], where the HMBS gene, which was stably expressed in bovine satellite cells during differentiation [7, 8], was used as a reference gene. Sequences of primers used in qPCR are described in Additional file 1.

### Total cellular protein extraction and Western blot analysis

Total cellular protein was isolated by incubating cells with the radioimmunoprecipitation assay (RIPA) buffer (50 mM Tris-HCl pH 7.4, 150 mM NaCl, 1 mM EDTA, 1% NP-40, 0.5% sodium deoxycholate, and 0.1% SDS) supplemented with a proteinase inhibitor cocktail on ice for 10 min, followed by centrifugation at 15,000 ×g and 4 °C for 30 min. For western blot analysis, 20 µg of total protein were separated by SDS-PAGE with 10% separating gel. After separation, proteins were transferred from the gel to a nitrocellulose membrane by electrophoresis. The membrane was first blocked with 5% non-fat milk in tris-buffered saline with 0.1% Tween 20 (TBST) at room temperature for 1 h and then incubated with a primary antibody at 4 °C overnight. The membrane was washed twice with TBST and then incubated with a secondary antibody at room temperature for 1 h. Two primary antibodies were used in this study. One was a ß-tubulin antibody (E7 from DSHB, Iowa City, IA, USA), and the other one was a TFAP4 antibody (sc-377,042 from Santa Cruz Biotechnology, Sana Cruz, CA, USA), both used at a 1:1000 dilution. The secondary antibody used in this study was the IRDye 800CW goat anti-mouse IgG secondary antibody (926-32210 from LI-COR Biosciences, Lincoln, NE, USA) used at a 1:15,000 dilution. Western blots were visualized using a LI-COR Odyssey Infrared Image System 9120, and fluorescence intensity was quantified using the Image Studio Lite software (LI-COR Biosciences).

### Immunocytochemistry and fusion index calculation

Cells were first fixed by incubating them with 4% formaldehyde in PBS at room temperature for 15 min, followed by two rinses with PBS. Cells were permeabilized by incubation with 0.25% Triton X-100 at room temperature for 10 min. Cells were then incubated with 1% BSA and 0.05% Tween-20 in PBS at room temperature for 1 h with gentle shaking to block nonspecific binding. Cells were incubated with an antibody for the myosin heavy chain (MHC) proteins (NA-4 from DSHB) at a 1:100 dilution in PBS overnight at 4 °C. After two washes with PBS, cells were incubated with an anti-mouse IgG FITC secondary antibody (MilliporeSigma) at a 1:200 dilution for 1 h at room temperature. Finally, cells were stained with 1 µg/mL of 4’,6-diamidino-2-phenylindole (DAPI) for 10 min at room temperature. The stained cells were imaged using a fluorescence microscope. The number of total nuclei and that of nuclei located in MHC-stained myofibers containing 3 or more nuclei were counted from randomly selected areas of images using the ImageJ software. The fusion index was calculated as the ratio of the number of nuclei within myofibers to that of total nuclei.

### Statistical analysis

Two-group comparisons were performed using the t-test in JMP Pro 16 (SAS, Cary, NC, USA) or GraphPad Prism 9 (San Diego, CA, USA). All data are expressed as means ± standard errors.

## Results

### Identification of H3K4me1-, H3K27ac-, and H3K27me3-marked genomic regions in proliferating and differentiating bovine satellite cells

To identify enhancers active and repressed in proliferating and differentiating bovine satellite cells, we immunoprecipitated the DNA regions associated with H3K4me1, K3K27ac, and H3K27me3 from two biological replicates of cells. We constructed 12 ChIP-seq libraries from the immunoprecipitated DNA and 4 libraries from the Input DNA. Sequencing of these libraries generated on average 60 million of 150-nucleotide reads per library (Additional file 2). Reads from all 16 libraries had a more than 90% mapping rate to the bovine genome (Additional file 2). All 12 ChIP-seq libraries had a unique mapping rate of greater than 70%. The 4 Input libraries had a unique mapping rate of less than 60%. Lower unique mapping rates of sequences from the Input libraries were probably caused by overamplification during library preparation. Assessing the quality of enrichment with the phantompeakqualtools program [21] indicated that all 12 ChIP-seq libraries had relative strand correlation (RSC) values > 0.8 and that 7 of them had normalized strand coefficient (NSC) values > 1.05 (Additional file 3), the threshold values set by the ENCODE and modENCODE consortia for high-quality ChIP-seq libraries [29]. Based on these assessments, all 12 ChIP-seq libraries had medium or high quality of enrichment (Additional file 3).

**Fig. 1:**
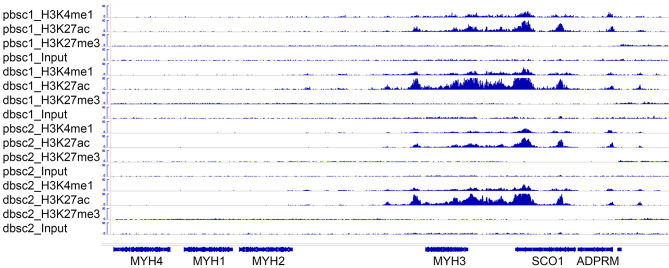
Examples of H3K4me1-, H3K27me3-, and H3K27ac-marked ChIP-seq peaks in proliferating bovine satellite cells (pbsc) and differentiating bovine satellite cells (dbsc). Shown at the left are the types of cells and histone marks characterized. Shown at the bottom are the genes associated with the peaks

Analyzing the ChIP-seq libraries against the corresponding Input libraries using the MACS program [20], we identified 43,936 to 99,968 H3K4me1-, H3K27ac- or H3K27me3-marked genomic regions or peaks (Table 1). Using the bedtools intersect program [22], we identified 84,641 overlapping H3K4me1 peaks, 64,995 overlapping H3K27ac peaks, and 50,956 overlapping H3K27me3 peaks between two biological replicates of proliferating bovine satellite cells and 80,780 overlapping H3K4me1 peaks, 61,552 overlapping H3K27ac peaks, and 59,174 overlapping H3K27me3 peaks between two biological replicates of differentiating bovine satellite cells (Table 1). Examples of these peaks and their associated genes (the myosin heavy chain gene cluster) are shown in Fig. 1. The complete lists of these peaks are shown in Additional files 4 and 5.

**Table 1:** Numbers of peaks identified in ChIP-seq libraries

Cells^1^	Library	Peak Type	Peaks of Biol. Rep. 1	Peaks of Biol. Rep. 2	Overlapping Peaks
pbsc	H3K4me1	broad	93,747	63,140	84,641
pbsc	H3K27ac	narrow	71,666	49,791	64,995
pbsc	H3K27me3	broad	58,958	44,937	50,956
dbsc	H3K4me1	broad	96,151	58,930	80,780
dbsc	H3K27ac	narrow	69,865	50,518	61,552
dbsc	H3K27me3	broad	99,968	43,936	59,174

^1^pbsc: proliferating bovine satellite cells; dbsc: differentiating bovine satellite cells

### Genomic distribution of H3K4me1, H3K27ac, and H3K27me3 peaks in proliferating and differentiating bovine satellite cells

**Fig. 2:**
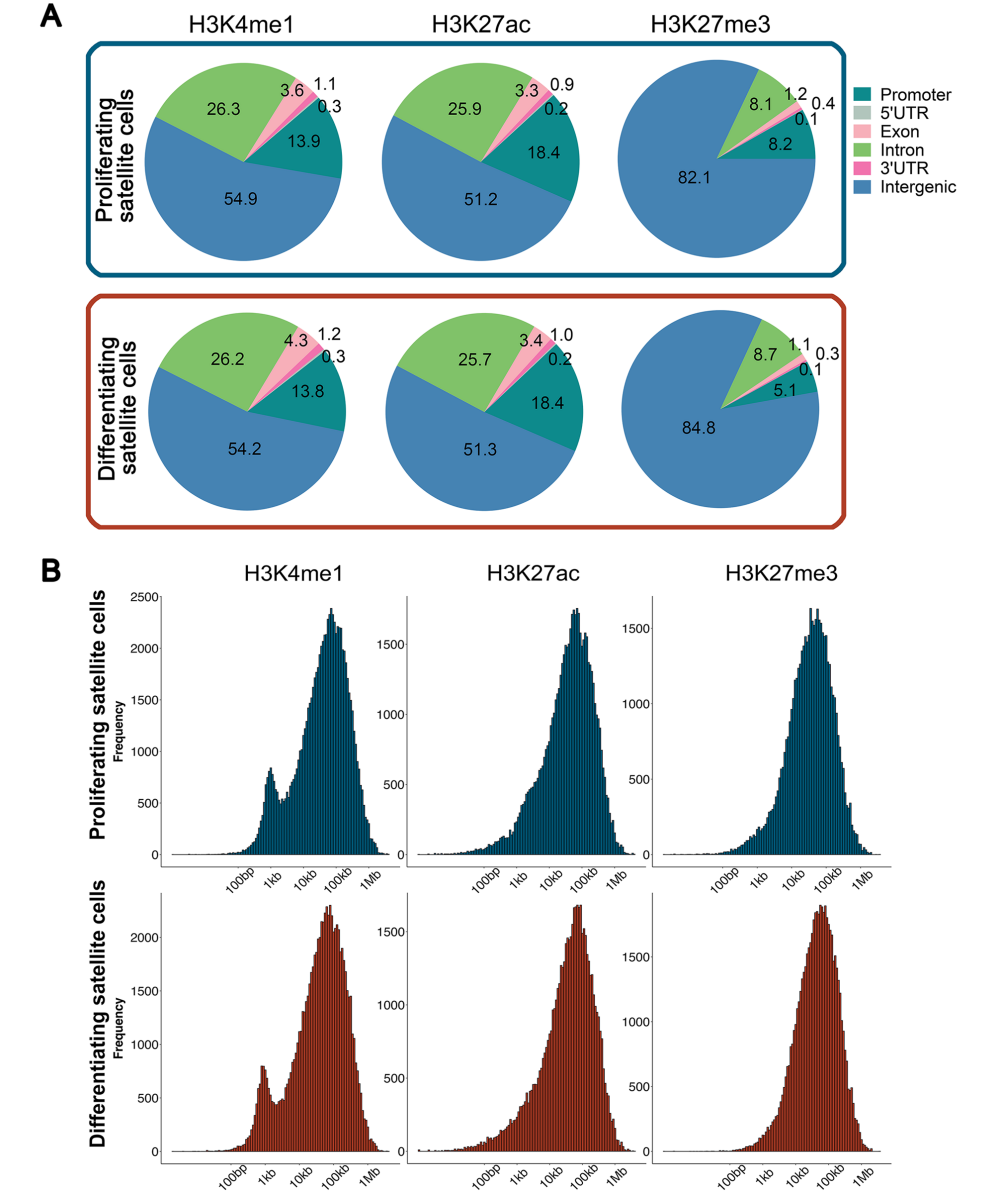
Genomic distribution of H3K4me1-, H3K27ac-, and H3K27me3-marked ChIP-seq peaks in proliferating and differentiating bovine satellite cells. **A** Percentage of peaks located in functionally different genomic regions; **B** Frequency of peaks located at different distances from the transcription start site (TSS)

More than 50% of H3K4me1 and H3K27ac peaks in proliferating or differentiating bovine satellite cells were located in the intergenic regions, approximately 25% of them in the introns, around 15% of them in the promoter regions, and less than 5% of them in the exons, 5’UTRs, or 3’UTRs (Fig. 2A). Notably, a much higher percentage (82%) of H3K27me3 peaks were located in the intergenic regions than that of H3K4me1 or H3K27ac peaks (Fig. 2A). In terms of distance from the transcription start site (TSS), both H3K27ac and H3K27me3 peaks concentrated at a long distance (∼ 80 kb) from the TSS, but H3K4me1 peaks concentrated at both a long distance (∼ 80 kb) and a short distance (∼ 800 bp) from the TSS (Fig. 2B). There was no apparent difference in the genomic distribution pattern of H3K4me1, H3K27ac, or H3K27me3 peaks between proliferating and differentiating bovine satellite cells (Fig. 2A and B).

### Identification of active and repressed enhancers in proliferating and differentiating bovine satellite cells

As mentioned in the section of Introduction, active enhancers are marked with both H3K4me1 and H3K27ac and repressed enhancers are often associated with H3K27me3 [30, 31]. By overlapping 84,641 H3K4me1-marked ChIP-seq peaks with 64,995 H3K27ac-marked peaks, we identified 56,973 active enhancers marked with both H3K4me1 and H3K27ac in proliferating bovine satellite cells (Additional file 4). Similarly, from 80,780 H3K4me1-marked and 61,552 H3K27ac-marked peaks, we identified 54,470 active enhancers marked with both H3K4me1 and H3K27ac in differentiating bovine satellite cells (Additional file 5). As described above, we identified 50,956 and 59,174 H3K27me3-marked genomic regions, i.e., repressed enhancers, in proliferating and differentiating bovine satellite cells, respectively (Additional files 4 and 5).

### Genes associated with active enhancers and repressed enhancers in proliferating and differentiating bovine satellite cells

Annotation of the ChIP-seq peaks with the closest genes indicated that the 56,793 H3K4me1- and H3K27ac-marked active and 50,956 H3K27me3-marked repressed enhancers in proliferating bovine satellite cells were associated with 9,053 and 6,859 genes, respectively (Additional file 4), and that the 54,771 H3K4me1- and H3K27ac-marked active and 59,174 H3K27me3-marked repressed enhancers in differentiating bovine satellite cells were associated with 8,761 and 6,580 genes, respectively (Additional file 5). Nearly a half of the genes associated with H3K4me1- and H3K27ac-marked active enhancers were also associated with H3K27me3-marked repressed enhancers in proliferating or differentiating bovine satellite cells (Additional files 4 and 5).

Notably, genes associated with active enhancers in proliferating bovine satellite cells included MYOD1 and MYF5, genes that define proliferating satellite cells [32, 33]; CCND1, CCND2, CCND3, CCNE2, CCNA2, CCNB1, and CCNB2, genes that regulate cell cycle [34]; and ID1, ID2, and ID3, genes that inhibit myoblast differentiation [35]. Genes associated with repressed enhancers in proliferating bovine satellite cells included MYOG, a widely-known master regulator of myoblast differentiation [4], and MYMK, a gene that is essential for myoblast fusion [36]. Genes associated with active enhancers in differentiating bovine satellite cells included MYOG, MYMK, and many genes (e.g., MYH3 and MYL1) encoding skeletal muscle contractile proteins [37]. Interestingly, genes associated with repressed enhancers in differentiating bovine satellite cells included CCND1, CCND2, CCNE1, and ID2, which, as mentioned above, were associated with active enhancers in proliferating cells. The complete lists of these genes are shown in Additional files 4 and 5.

Functional enrichment analyses of genes associated with H3K4me1- and H3K27ac-marked active enhancers in proliferating bovine satellite cells showed that regulation of protein catabolic process, protein folding, proteasome complex, vesicle coat, catalytic activity, and actin filament binding were among the biological processes, cellular components, or molecular functions significantly enriched in these genes (Table 2). Functional enrichment analyses of genes associated with H3K4me1- and H3K27ac-marked active enhancers in differentiating bovine satellite cells showed that autophagosome assembly, muscle structure development, Z disc, ubiquitin conjugating enzyme activity, and transcription factor binding were among the biological processes, cellular components, or molecular functions significantly enriched in these genes (Table 3).

**Table 2:** Top 5 GO terms enriched in genes associated with H3K4me1- and H3K27ac-marked active enhancers in proliferating bovine satellite cells

GO category	GO term	Count of genes	Fold enrichment	*P*-value	FDR
Biological process	regulation of protein catabolic process (GO:0042176)	58	2.0	0.000	0.003
	regulation of proteasomal protein catabolic process (GO:0061136)	37	1.9	0.004	0.046
	protein folding (GO:0006457)	84	1.8	0.000	0.002
	vacuole organization (GO:0007033)	49	1.8	0.003	0.038
	Golgi vesicle transport (GO:0048193)	115	1.8	0.000	0.000
Cellular component	proteasome complex (GO:0000502)	33	2.0	0.005	0.033
	vesicle coat (GO:0030120)	33	1.9	0.007	0.045
	coated vesicle membrane (GO:0030662)	39	1.9	0.003	0.025
	focal adhesion (GO:0005925)	45	1.9	0.001	0.013
	membrane coat (GO:0030117)	39	1.9	0.003	0.026
Molecular function	catalytic activity, acting on RNA (GO:0140098)	164	1.5	0.000	0.001
	catalytic activity, acting on a nucleic acid (GO:0140640)	251	1.5	0.000	0.000
	actin filament binding (GO:0051015)	108	1.5	0.002	0.024
	GTPase activity (GO:0003924)	125	1.5	0.001	0.013
	actin binding (GO:0003779)	146	1.5	0.000	0.008

**Table 3:** Top 5 GO terms enriched in genes associated with H3K4me1- and H3K27ac-marked active enhancers in differentiating bovine satellite cells

GO category	GO term	Count of genes	Fold enrichment	*P*-value	FDR
Biological process	autophagosome assembly (GO:0000045)	34	2.0	0.003	0.039
	muscle structure development (GO:0061061)	49	1.9	0.001	0.018
	vacuole organization (GO:0007033)	51	1.9	0.001	0.013
	macroautophagy (GO:0016236)	48	1.8	0.002	0.023
	regulation of RNA splicing (GO:0043484)	51	1.8	0.002	0.024
Cellular component	proteasome accessory complex (GO:0022624)	19	2.6	0.006	0.037
	proteasome regulatory particle (GO:0005838)	19	2.6	0.006	0.037
	Z disc (GO:0030018)	30	2.2	0.002	0.018
	replication fork (GO:0005657)	24	2.1	0.008	0.049
	actin filament bundle (GO:0032432)	24	2.1	0.008	0.049
Molecular function	ubiquitin conjugating enzyme activity (GO:0061631)	33	2.1	0.003	0.028
	ubiquitin-like protein conjugating enzyme activity (GO:0061650)	34	2.0	0.003	0.032
	transcription factor binding (GO:0008134)	53	1.8	0.002	0.026
	S-adenosylmethionine-dependent methyltransferase activity (GO:0008757)	66	1.7	0.001	0.016
	methyltransferase activity (GO:0008168)	86	1.59	0.001	0.013

Functional enrichment analyses of genes associated with H3K27me3-marked repressed enhancers in proliferating bovine satellite cells showed that regulation of blood circulation, axon terminus, neuron projection terminus, chemokine binding, and cytokine binding were among the biological processes, cellular components, or molecular functions significantly enriched in these genes (Table 4). Functional enrichment analyses of genes associated with H3K27me3-marked repressed enhancers in differentiating bovine satellite cells revealed that regulation of blood circulation, eye development, receptor complex, axon, peptide hormone binding, and chemokine binding were among the biological processes, cellular components, or molecular functions significantly enriched in these genes (Table 5).

**Table 4:** Top 5 GO terms enriched in genes associated with H3K27me3-marked repressed enhancers in proliferating bovine satellite cells

GO category	GO term	Count of genes	Fold enrichment	*P*-value	FDR
Biological process	regulation of systemic arterial blood pressure (GO:0003073)	19	2.7	0.002	0.030
	regulation of blood circulation (GO:1,903,522)	24	2.5	0.001	0.024
	regulation of system process (GO:0044057)	35	2.3	0.000	0.007
	circulatory system process (GO:0003013)	45	2.2	0.000	0.004
	blood circulation (GO:0008015)	43	2.1	0.000	0.005
Cellular component	axon terminus (GO:0043679)	27	2.4	0.001	0.015
	neuron projection terminus (GO:0044306)	27	2.3	0.002	0.022
	receptor complex (GO:0043235)	128	1.8	0.000	0.000
	extrinsic component of plasma membrane (GO:0019897)	51	1.8	0.002	0.021
	adherens junction (GO:0005912)	48	1.8	0.002	0.025
Molecular function	chemokine binding (GO:0019956)	21	2.5	0.003	0.036
	C-C chemokine binding (GO:0019957)	19	2.4	0.005	0.047
	cytokine binding (GO:0019955)	49	2.1	0.000	0.002
	growth factor binding (GO:0019838)	29	2.1	0.004	0.039
	cytokine receptor activity (GO:0004896)	48	2.0	0.000	0.006

**Table 5:** Top 5 GO terms enriched in genes associated with H3K27me3-marked repressed enhancers in differentiating bovine satellite cells

GO category	GO term	Count of genes	Fold enrichment	*P*-value	FDR
Biological process	regulation of blood circulation (GO:1,903,522)	22	2.3	0.003	0.045
	regulation of system process (GO:0044057)	33	2.3	0.001	0.012
	eye development (GO:0001654)	32	2.2	0.001	0.018
	visual system development (GO:0150063)	32	2.2	0.001	0.017
	sensory system development (GO:0048880)	32	2.2	0.001	0.017
Cellular component	receptor complex (GO:0043235)	110	1.6	0.000	0.002
	axon (GO:0030424)	68	1.6	0.002	0.033
	dendritic tree (GO:0097447)	69	1.6	0.002	0.037
	dendrite (GO:0030425)	69	1.6	0.002	0.035
	somatodendritic compartment (GO:0036477)	84	1.6	0.001	0.025
Molecular function	peptide hormone binding (GO:0017046)	17	2.6	0.004	0.045
	chemokine binding (GO:0019956)	20	2.4	0.003	0.040
	cytokine binding (GO:0019955)	45	2.0	0.000	0.006
	peptide receptor activity (GO:0001653)	59	2.0	0.000	0.002
	cytokine receptor activity (GO:0004896)	45	1.9	0.001	0.014

### Transcription factor binding sites enriched in active enhancers and repressed enhancers in proliferating and differentiating bovine satellite cells

**Fig. 3:**
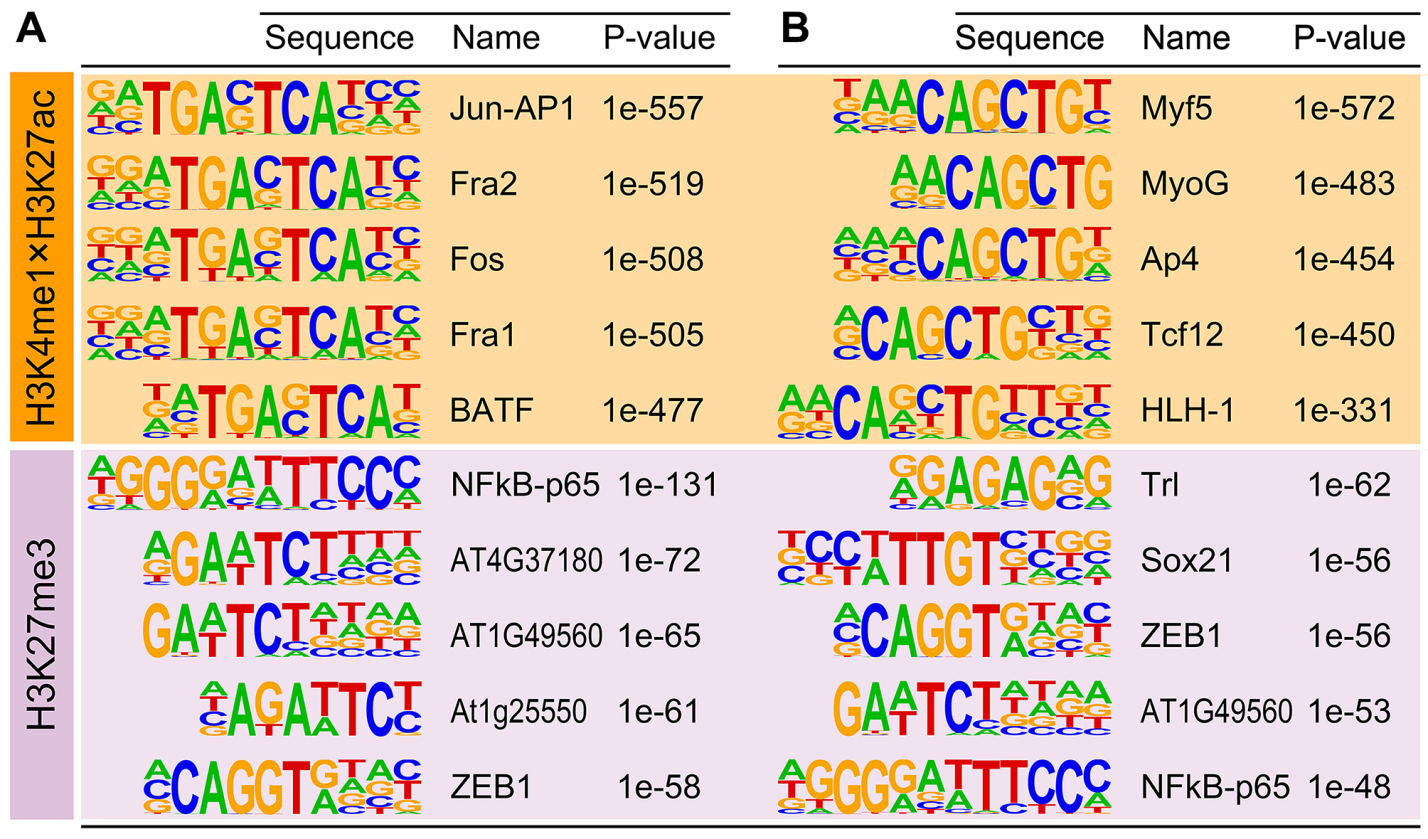
Top 5 motifs (i.e., transcription factor binding sites) enriched in H3K4me1-, H3K27ac-, and H3K27me3-marked genomic sequences. **A** Top 5 motifs enriched in proliferating bovine satellite cells. **B** Top 5 motifs enriched in differentiating bovine satellite cells. Motifs were identified using the Homer program and ranked according to the *P*-value

Based on the motif enrichment analyses, H3K4me1- and H3K27ac-marked active enhancers and H3K27me3-marked repressed enhancers in proliferating bovine satellite cells were each enriched with binding sites for many transcription factors (Additional files 6–9). The top 5 motifs enriched in H3K4me1- and H3K27ac-marked active enhancers in proliferating bovine satellite cells were binding sites for the transcription factors Jun-AP1, Fra2, Fos, Fra1, and BATF (Fig. 3A), all of which are members of the AP-1/ATF superfamily of transcription factors [38]. The top 5 motifs enriched in H3K4me1- and H3K27ac-marked active enhancers in differentiating bovine satellite cells were binding sites for the transcription factors Myf5, MyoG, Ap4, Tcf12, and HLH-1 (Fig. 3B). The top 5 motifs enriched in H3K27me3-marked repressed enhancers in proliferating bovine satellite cells were binding sites for the transcription factors NFkB-p65, ZEB1, AT4G37180, AT1G49560, and At1g25550 (Fig. 3A), and the top 5 motifs enriched in H3K27me3-marked repressed enhancers in differentiating bovine satellite cells were binding sites for the transcription factors Trl, Sox21, ZEB1, AT1G49560, and NFkB-p65 (Fig. 3B).

### Super-enhancers in proliferating and differentiating bovine satellite cells

**Fig. 4:**
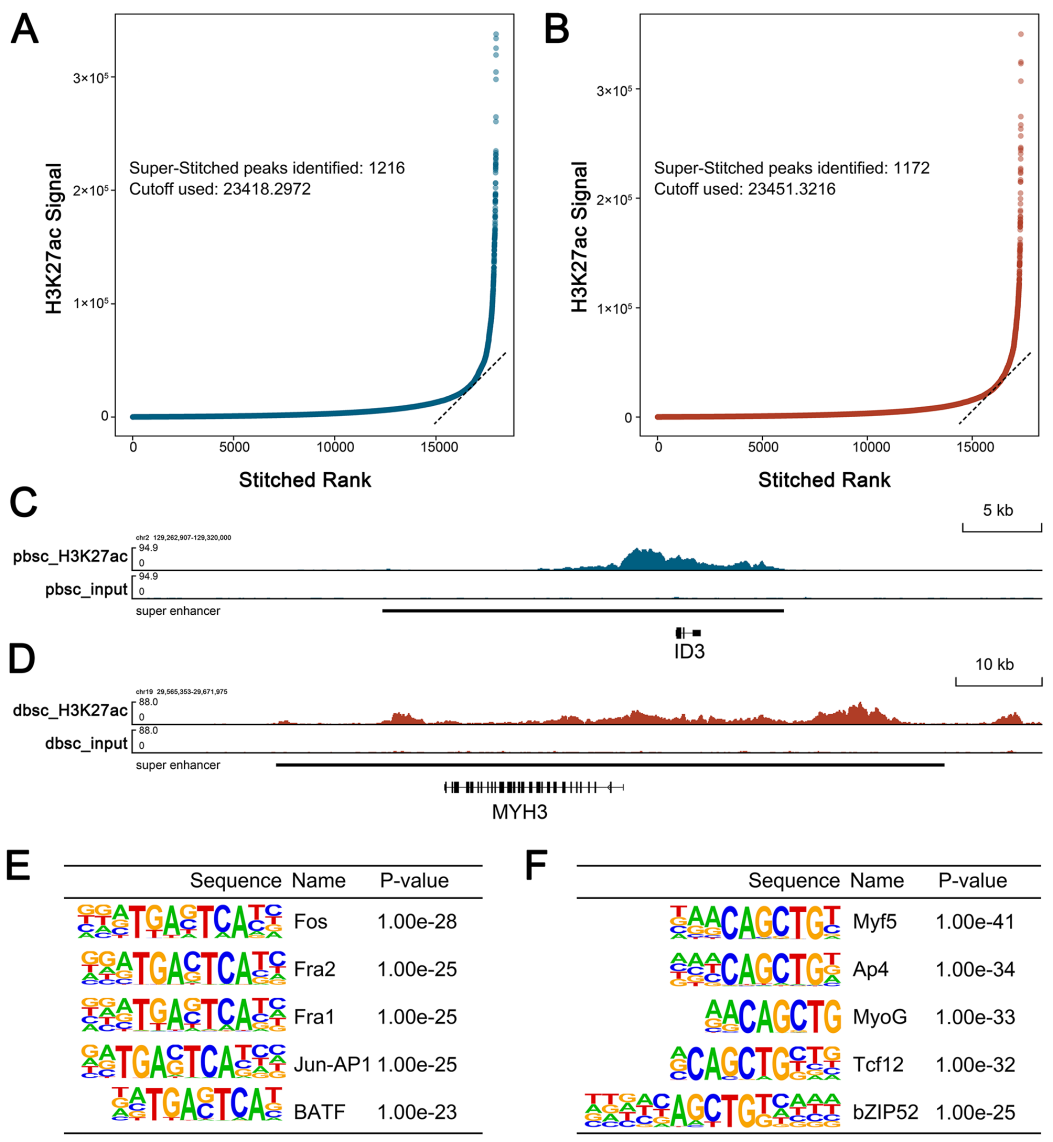
Super-enhancers in proliferating and differentiating bovine satellite cells. Super-enhancers were identified by analyzing H3K27ac-marked active enhancers using the ROSE program. **A, B** Numbers of super-enhancers in proliferating and differentiating bovine satellite cells, respectively. **C**, **D** Examples of super-enhancers (highlighted with a thick black line) in proliferating and differentiating bovine satellite cells, respectively. **E, F** Top 5 motifs enriched in super-enhancers in proliferating and differentiating bovine satellite cells, respectively

Super-enhancers are genomic regions containing multiple enhancers and multiple binding sites for transcription factors [25, 39]. Using the ROSE program [25], we identified 1,216 and 1,176 super-enhancers, from 64,995 and 61,552 H3K27ac-marked active enhancers in proliferating and differentiating bovine satellite cells, respectively (Fig. 4A and B). Two examples of these super-enhancers are shown in Fig. 4C and D. The complete lists of these super-enhancers are shown in Additional files 10 and 11. Motif enrichment analyses showed that the top 5 motifs enriched in the super-enhancers in proliferating bovine satellite cells were binding sites for members of the AP-1/ATF superfamily of transcription factors, including Fos, Fra1, Fra2, Jun-AP1, and BATF (Fig. 4E) and that the top 5 motifs enriched in the super-enhancers in differentiating bovine satellite cells included binding sites for Myf5, Ap4, MyoG, Tcf12, and bZIP52 (Fig. 4F).

Functional enrichment analyses of genes associated with the super-enhancers in proliferating bovine satellite cells revealed that positive regulation of myeloid cell differentiation, actin filament depolymerization, actin cortical patch, focal adhesion, collagen binding, and extracellular matrix binding were among the biological processes, cellular components, or molecular functions enriched in these genes (Table 6). Functional enrichment analyses of genes associated with the super-enhancers in differentiating bovine satellite cells revealed that negative regulation of angiogenesis, muscle cell development, Z disc, I band, collagen binding, and cadherin binding were among the biological processes, cellular components, or molecular functions enriched in these genes (Table 7).

**Table 6:** Top 5 GO terms enriched in genes associated with super-enhancers in proliferating bovine satellite cells

GO category	GO term	Count of genes	Fold enrichment	*P*-value	FDR
Biological process	positive regulation of myeloid cell differentiation (GO:0045639)	4	17.1	0.000	0.009
	actin filament depolymerization (GO:0030042)	6	16.0	0.000	0.001
	leucine transport (GO:0015820)	3	16.0	0.003	0.040
	liver development (GO:0001889)	4	10.7	0.001	0.025
	response to reactive oxygen species (GO:0000302)	6	9.2	0.000	0.005
Cellular component	actin cortical patch (GO:0030479)	6	9.2	0.000	0.002
	focal adhesion (GO:0005925)	24	8.7	0.000	0.000
	cell-substrate junction (GO:0030055)	24	8.1	0.000	0.000
	stress fiber (GO:0001725)	9	8.0	0.000	0.000
	actomyosin (GO:0042641)	9	7.7	0.000	0.000
Molecular function	collagen binding (GO:0005518)	5	7.1	0.002	0.029
	extracellular matrix binding (GO:0050840)	5	5.9	0.003	0.041
	cadherin binding (GO:0045296)	16	5.4	0.000	0.000
	glycosaminoglycan binding (GO:0005539)	10	5.2	0.000	0.002
	heparin binding (GO:0008201)	6	4.7	0.003	0.041

**Table 7:** Top 5 GO terms enriched in genes associated with super-enhancers in differentiating bovine satellite cells

GO category	GO term	Count of genes	Fold enrichment	*P*-value	FDR
Biological process	negative regulation of angiogenesis (GO:0016525)	5	11.1	0.000	0.013
	actin filament depolymerization (GO:0030042)	4	11.1	0.001	0.034
	muscle cell development (GO:0055001)	14	8.6	0.000	0.000
	activin receptor signaling pathway (GO:0032924)	5	8.5	0.001	0.025
	striated muscle cell development (GO:0055002)	9	8.3	0.000	0.001
Cellular component	Z disc (GO:0030018)	14	8.6	0.000	0.000
	I band (GO:0031674)	14	8.4	0.000	0.000
	focal adhesion (GO:0005925)	21	7.9	0.000	0.000
	cell-substrate junction (GO:0030055)	22	7.7	0.000	0.000
	sarcomere (GO:0030017)	19	7.5	0.000	0.000
Molecular function	collagen binding (GO:0005518)	5	7.4	0.001	0.028
	cadherin binding (GO:0045296)	14	4.9	0.000	0.000
	actin binding (GO:0003779)	50	4.4	0.000	0.000
	transmembrane receptor protein kinase activity (GO:0019199)	14	4.4	0.000	0.001
	phosphatidylinositol bisphosphate binding (GO:1,902,936)	8	4.3	0.001	0.024

### TFAP4 binding sites are present in many active enhancers in differentiating bovine satellite cells

**Fig. 5:**
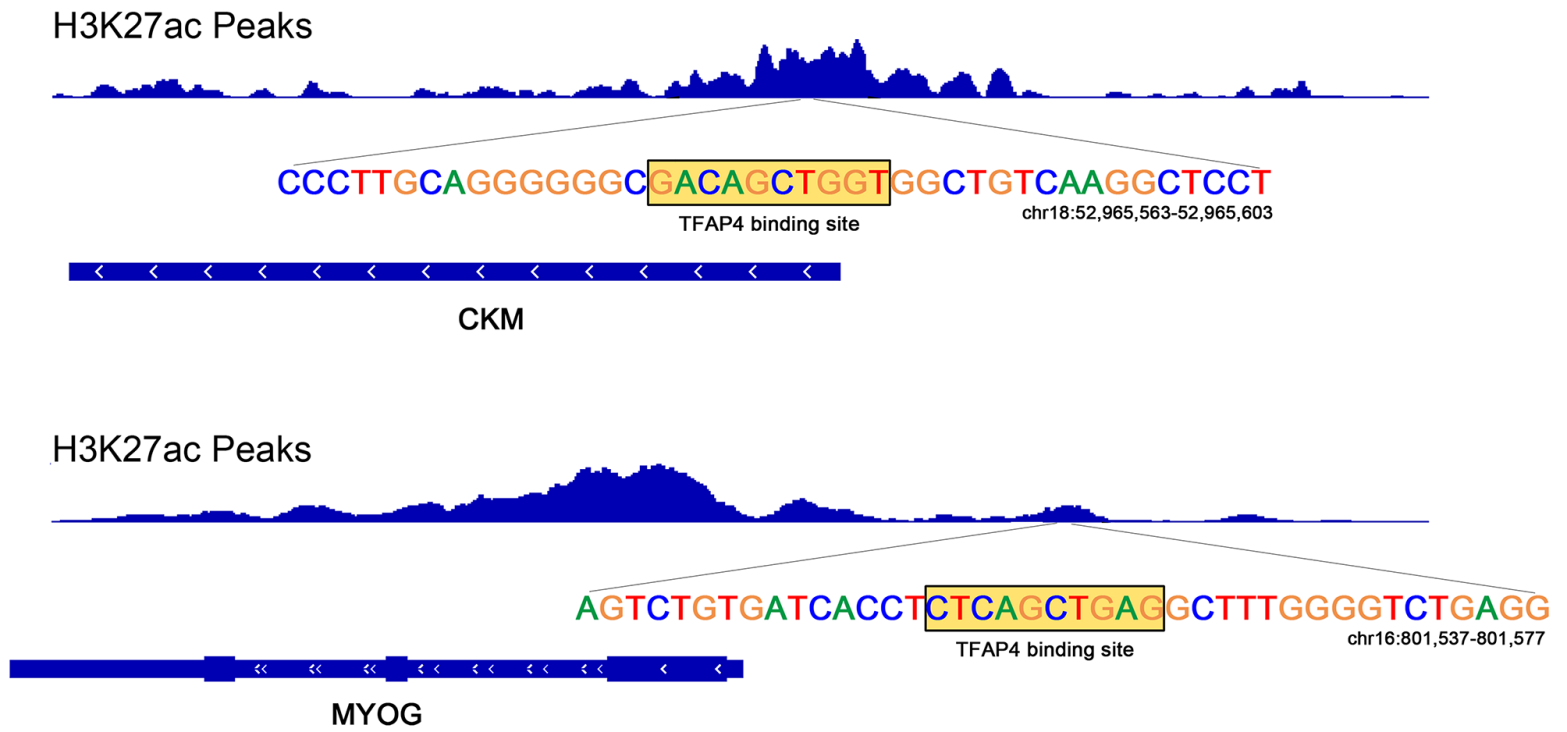
Examples of TFAP4 binding sites in H3K27ac-marked active enhancers in differentiating bovine satellite cells. TFAP4 binding sites were identified using the position weight matrix MA1570.1 and MA0691.1. Shown are H3K27ac peaks, 40-nucleotide sequences containing the TFAP4 binding sites, and associated genes

The motif enrichment analyses of both active enhancers and super-enhancers in bovine satellite cells indicated that gene expression during bovine satellite cell differentiation is controlled by many transcription factors in addition to MYOG. One of these additional transcription factors is Ap4, also known as TFAP4 (Figs. 3B and 4F), whose role in satellite cell or myoblast differentiation was previously unknown. Using the FIMO tool [22], we located 6,762 TFAP4 binding sites in H3K27ac-marked active enhancers in differentiating bovine satellite cells (Additional file 12). Figure 5 shows two examples of TFAP4 binding sites associated with the MYOG and CKM genes, the latter of which encodes a creatine kinase important for the generation of ATP for skeletal muscle contraction [40]. The 6,762 TFAP4 binding sites were associated with 3,157 genes (Additional file 12). Functional enrichment analyses indicated anatomical structure morphogenesis, cellular response to an organic substance, tissue development, regulation of intracellular signal transduction, response to endogenous stimulus, muscle organ development, muscle tissue development, and striated muscle hypertrophy were among the functional terms enriched in these TFAP4 binding site-associated genes.

### TFAP4 knockdown inhibited the differentiation and fusion of bovine satellite cells into myofibers

**Fig. 6:**
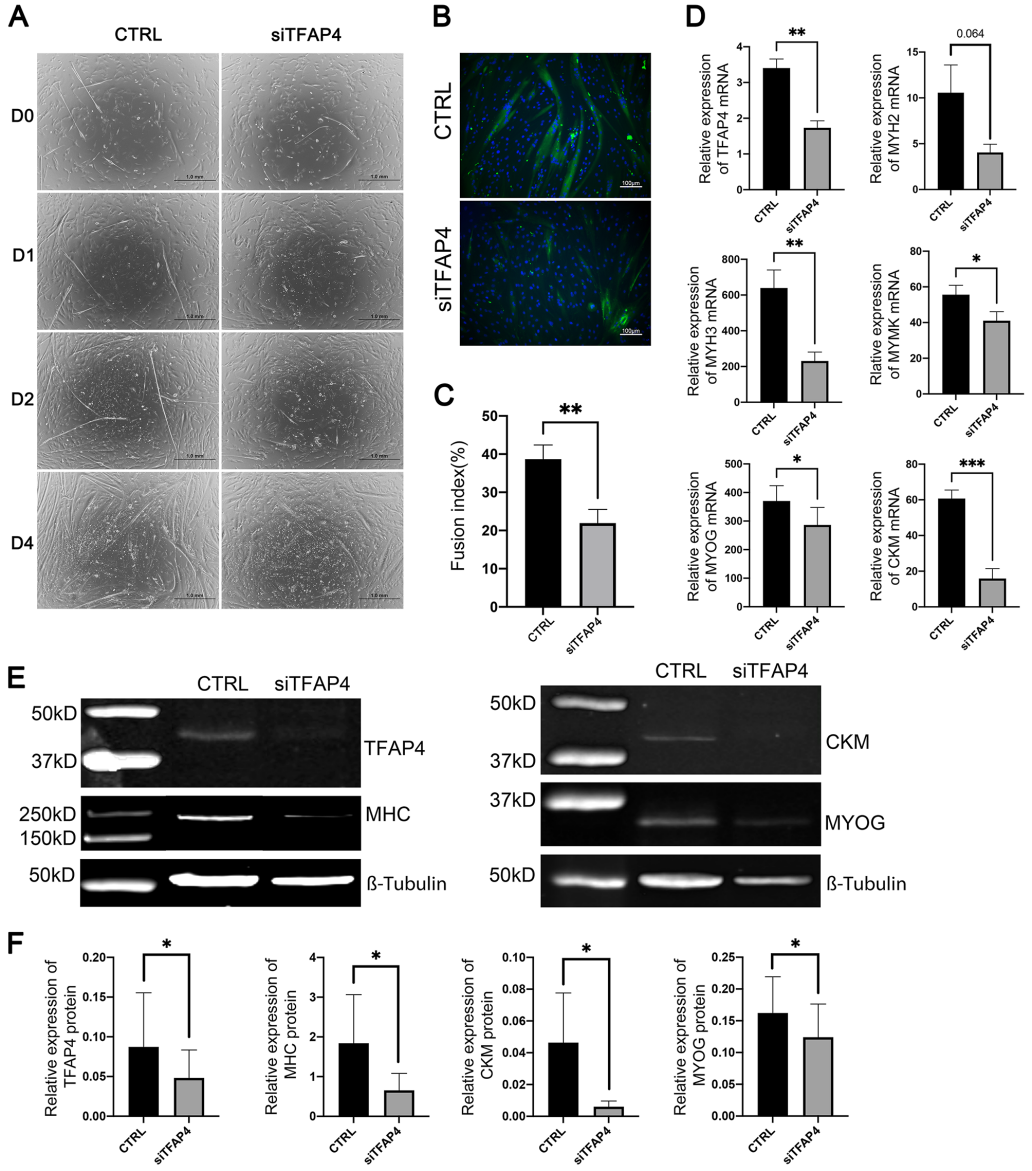
Effect of siRNA-mediated knockdown of TFAP4 on differentiation of bovine satellite cells. Cells were transfected with a negative control siRNA (CTRL) or a bovine TFAP4 mRNA-specific siRNA (siTFAP4) and then induced to differentiate into myofibers for 4 days. **A** Representative images of cells on days 0, 1, 2, and 4 of differentiation. **B** Representative images of immunostained cells on day 4 of differentiation. Staining of myosin heavy chain protein is shown in green color, and staining of nuclei is shown in blue color. **C** Fusion index of cells on day 4 of differentiation. **P < 0.02 (*n* = 4). **D** Quantification of TFAP4, MYH2, MYH3, MYOG, CKM, and MYMK mRNAs. *P < 0.05, **P < 0.002, ***P < 0.001 (*n* = 4). *P* = 0.064 for MYH2. **E** Western blot analyses of TFAP4, myosin heavy chain (MHC), CKM, and MYOG proteins. β-Tubulin was analyzed as a loading control. Shown are cropped representative western blots. Full-length western blots are presented in Additional file 13. **F** Quantitative analysis of western blots. *P < 0.05 (*n* = 3)

To investigate the role of TFAP4 in the differentiation of bovine satellite cells into myofibers, we knocked down the expression of TFAP4 in these cells using a bovine TFAP4-specific siRNA. Compared to bovine satellite cells transfected with a negative control siRNA, bovine satellite cells transfected with a bovine TFAP4 mRNA-specific siRNA showed delayed formation of myofibers during induced differentiation (Fig. 6A). Furthermore, myofibers formed from bovine satellite cells transfected with the TFAP4 siRNA were fewer and smaller than in formed from those transfected with the control siRNA (Fig. 6A). Myofibers formed from cells transfected with the TFAP4 siRNA also showed weaker staining for myosin heavy chain proteins than myofiber formed from cells transfected with the control siRNA (Fig. 6B). The fusion index for cells transfected with the TFAP4 siRNA was much smaller than that for those transfected with the control siRNA (*P* < 0.05, Fig. 6C). The expression levels of TFAP4 mRNA (Fig. 6D) and protein (Fig. 6E and F) in cells transfected with the TFAP4 siRNA were 50% less than those in those transfected with the control siRNA (*P* < 0.05), indicating effective knockdown of TFAP4 expression by the transfected TFAP4 siRNA. These data together indicated that siRNA-induced knockdown of TFAP4 inhibited the capacity of bovine satellite cells to differentiate and fuse into myofibers.

To further determine the effect of TFAP4 knockdown on the differentiation and fusion of bovine satellite cells into myofibers, we quantified the expression levels of selected marker genes of myofibers, including MYH2, MYH3, MYMK, MYOG, and CKM. As shown in Fig. 6D, the mRNA levels of these five marker genes were significantly lower in cells transfected with the TFAP4 siRNA than in cells transfected with the control siRNA (*P* < 0.05). We also determined the effect of TFAP4 knockdown on the expression of MHC (myosin heavy chain), MYOG, and CKM genes at the protein level. As shown in Fig. 6E and F, the protein levels of these myofiber markers were 50% less in TFAP4-knocked down cells than in control cells (*P* < 0.05). These expression data were consistent with the morphological data (Fig. 6A), indicating that TFAP4 knockdown inhibited the differentiation and fusion of bovine satellite cells into myofibers.

### TFAP4 overexpression increased the expression of myofiber marker genes in bovine satellite cells during differentiation

**Fig. 7:**
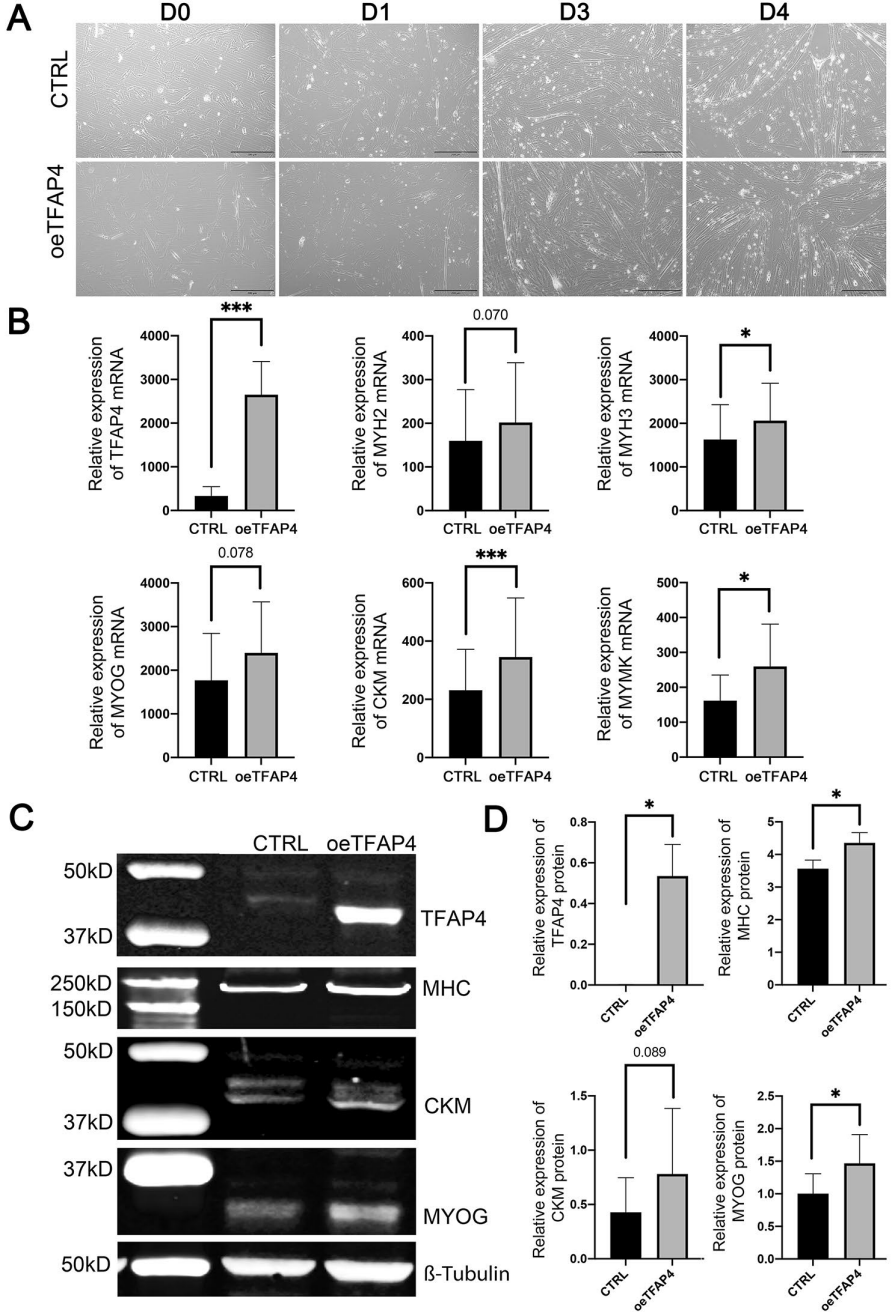
Effect of TFAP4 overexpression on differentiation of bovine satellite cells. Cells were transfected with a TFAP4 expression plasmid (oeTFAP4) or a negative control plasmid (CTRL) and induced to differentiate into myofibers for 4 days. **A** Representative images of cells on days 0, 1, 3 and 4 of differentiation. **B** mRNA expression levels of myofiber marker genes MYH2, MYH3, MYOG, CKM, and MYMK as well as TFAP4. *P < 0.05, ***P < 0.001 (*n* = 6). *P* = 0.070 and 0.078 for MYH2 and MYOG, respectively. **C** Western blot analyses of TFAP4, MHC, CKM, and MYOG proteins. β-Tubulin was detected as a loading control. Shown are cropped representative western blots. Full-length western blots are presented in Additional file 13. **D** Quantitative analysis of western blots. *P < 0.05 (*n* = 3) for TFAP4, CKM, and MYOG; *P* = 0.089 for CKM

We also determined the effect of TFAP4 overexpression on the differentiation and fusion of bovine satellite cells into myofibers. While bovine satellite cells transfected with the TFAP4 expression plasmid were morphologically not different from those transfected with a negative control plasmid during the 4-day differentiation (Fig. 7A), the mRNA levels of myofiber marker genes MYH3, CKM, and MYMK were higher (*P* < 0.05) in TFAP4-overexpressed cells than in control cells, and the mRNA levels of MYH2 and MYOG tended to be higher (*P* < 0.1) in TFAP4-overexpressed cells than in control cells (Fig. 7B). Western blot analyses showed that the expression levels of MHC, CKM, and MYOG proteins were higher or tended to be higher in TFAP4-overexpressed cells than in control cells (Fig. 7C and D). Transfection of bovine satellite cells with a TFAP4-expression plasmid increased (*P* < 0.05) the expression of TFAP4 at both the mRNA (Fig. 7B) and protein (Fig. 7C and D) levels. These data indicated that TFAP4 overexpression had a moderate stimulatory effect on the differentiation and fusion of bovine satellite cells into myofibers.

## Discussion

In this study, we aimed to identify the enhancers and the transcription factors binding to these enhancers that control gene expression during bovine satellite cell proliferation and differentiation. We started this study by performing a genome-wide mapping of the DNA sequences associated with the histone modifications H3K4me1, H3K27ac, and H3K27me3, which mark different types of enhancers [31]. Using ChIP-seq, we identified 50,000 to 80,000 genomic regions associated with H3K4me1, H3K27ac, or H3K27me3 from proliferating or differentiating bovine satellite cells. These numbers are similar to the numbers of enhancers isolated in various cell and tissue types [31]. Much more H3K4me1-marked genomic regions than H3K27ac- or H3K27me3-marked enhancers were identified from both proliferating and differentiating bovine satellite cells. This is consistent with the facts that H3K4me1 marks both active and poised enhancers, that H3K27ac marks active enhancers only, and that H3K27me3 marks a subset of poised enhancers or repressed enhancers [31]. Our genomic distribution analyses of these enhancers indicated that most of them are located in the intergenic or intronic regions, consistent with the general location of enhancers in the genome [41]. Our data also showed that the genomic locations of these enhancers are not different between proliferating and differentiating bovine satellite cells, suggesting that the genomic location of enhancers in bovine satellite cells is not affected by the developmental stage of these cells.

In this study, we found that nearly 50% of the genes associated with H3K27ac-marked active enhancers were also associated with H3K27me3-marked repressed enhancers. This result suggests that in bovine satellite cells a significant number of genes are under the control of both transcriptional activators and repressors. Through functional enrichment analyses, we found that genes associated with H3K27ac-marked active enhancers in proliferating bovine satellite cells were enriched with those functioning in gene transcription, RNA splicing, and translation, that genes associated with H3K27me3-marked repressed enhancers in proliferating bovine satellite cells were enriched with those involved in the vascular process and synaptic formation, that genes associated with H3K27ac-marked active enhancers in differentiating bovine satellite cells were enriched with those functioning in skeletal muscle development and encoding the contractile proteins, and that genes associated with H3K27me3-amrked repressed enhancers in differentiating bovine satellite cells were enriched with those involved in BMP singling and digestive tract development. These findings imply that in proliferating bovine satellite cells expression of genes essential for cell proliferation and survival is enhanced while in differentiating bovine satellite cells expression of genes essential for satellite cell differentiation and fusion into myofibers is enhanced and that expression of genes not essential for the proliferation or differentiation of bovine satellite cells is inhibited. These implications are largely consistent with gene expression profiles in proliferating and differentiating bovine satellite cells determined by RNA sequencing [8].

In this study, we found that the binding sites for the AP-1/ATF superfamily of transcription factors were the top motifs enriched in active enhancers and super-enhancers in proliferating bovine satellite cells. This finding suggests that the AP-1/ATF family of transcription factors play a central role in driving gene expression in proliferating bovine satellite cells. This finding is novel because MyoD1 and Myf5 are widely considered as the major transcription factors that drive the expression of genes involved in cell cycle in proliferating myoblasts [6, 42, 43]. The AP-1/ATF family of transcription factors have been shown to play a major role in the proliferation of various cell types [44]; so, it should not be surprising if these factors are also the major transcription factors that regulate the proliferation of satellite cells.

In this study, we found that both active enhancers and super-enhancers in differentiating bovine satellite cells were enriched with binding sites for many transcription factors in addition to MyoG, which is known as a master transcriptional regulator of myoblast differentiation [42, 45, 46]. These additional transcription factors included Myf5, Tcl12, TFAP4, and AP-1. Myf5 is known for its role in the determination and proliferation of myoblasts or satellite cells [47]. Enrichment of the Myf5 binding sites in active enhancers and super-enhancers in differentiating bovine satellite cells suggests that this transcription factor might also play a role in the differentiation of satellite cells. Similarly, enrichment of AP-1 binding sites in active enhancers and super-enhancers in differentiating bovine satellite cells suggests that this transcription factor might play a role in both the proliferation and differentiation of bovine satellite cells.

TFAP4 is a transcription factor known for its role in epithelial-mesenchymal transition and cancer [48–51]. Enrichment of the TFAP4 binding site in active enhancers and super-enhancers in differentiating bovine satellite cells suggests that TFAP4 might also play a role in satellite cell differentiation. This study found that many bovine myogenic genes contain TFAP4 binding sites and that siRNA-induced knockdown of TFAP4 in bovine satellite cells inhibited the formation of myofibers and the expression of myogenic genes containing TFAP4 binding sites during differentiation. These results suggest that TFAP4 plays a critical role in satellite cell differentiation by directly controlling the expression of myogenic genes. In this study, we found that TFAP4 overexpression, however, did not cause as significant an effect as TFAP4 knockdown on the differentiation and fusion of bovine satellite cells into myofibers. This difference suggests that the expression level of endogenous TFAP4 in bovine satellite cells might be sufficient for affecting gene expression and cell differentiation and that the activity (i.e., post-translational modification) rather than the expression level of TFAP4 might determine its role in the differentiation and fusion of bovine satellite cells into myofibers.

In this study, we found that H3K27me3-marked repressed enhancers in both proliferating and differentiating bovine satellite cells were enriched with binding sites for NF-kB, a transcription factor known for its role in immunity, inflammation, cell proliferation, cell survival, and cell differentiation [52], and ZEB1, a transcription factor known for its role in epithelial-mesenchymal transition in cells [53]. This finding suggests that genes normally targeted by NF-kB and ZEB1 are repressed in both proliferating and differentiating bovine satellite cells by PRC2, the protein complex that deposits H3K27me3 [54]. In mammalian cells how PRC2 is recruited to target genomic regions is not clear, and a consensus response element has not been identified for mammalian PRC2 [55]. Enrichment of NF-kB and ZEB1 binding sites in H3K27me3-marked genomic regions suggests the possibility that these two transcription factors might be involved in recruiting the PRC2 complex to its target DNA sequences in bovine satellite cells.

**Fig. 8:**
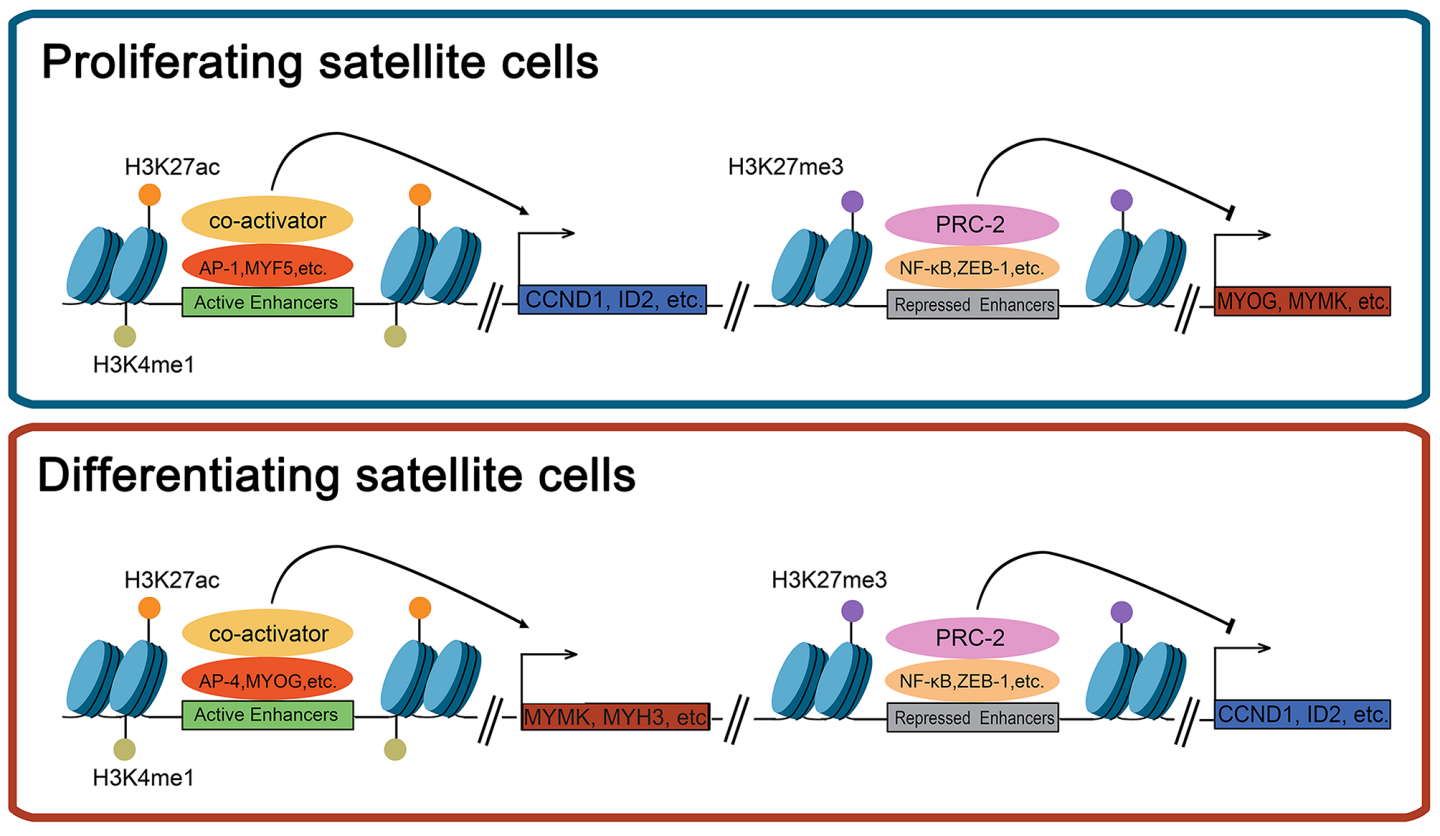
A model of transcriptional regulation in bovine satellite cells. In proliferating bovine satellite cells, genes that regulate cell cycle (e.g., CCND1) and genes that inhibit myoblast differentiation (e.g., ID1) are activated by the AP-1 family transcription factors and the myogenic regulatory factor 5 (MYF5) binding to active enhancers marked with H3K4me1 and H3K27ac, whereas genes that stimulate myoblast differentiation (e.g., MYOG) and fusion (e.g., MYMK) are repressed by the PRC-2 complex potentially recruited to repressed enhancers by the transcription factors NF-kB and ZEB-1. In differentiating bovine satellite cells, genes that encode proteins important for muscle contraction (e.g., MYH3) and for myoblast fusion (e.g., MYMK) are activated by the transcription factors MYOG and TFAP4 binding to active enhancers whereas genes that stimulate cell proliferation (e.g., CCND1) and inhibit myoblast differentiation (e.g., ID2) are repressed by the PRC-2 complex

## Conclusions

In summary, we have identified thousands of active and repressed enhancers and genes likely controlled by these enhancers in proliferating and differentiating bovine satellite cells. Our data suggest that many transcription factors including the AP-1 family of transcription factors control the expression of genes in proliferating bovine satellite cells and that besides MyoG, many other transcription factors such as TFAP4 control the expression of genes in differentiating bovine satellite cells. We have also found that in proliferating bovine satellite cells, genes stimulating cell proliferation such as CCND1 and genes inhibiting myoblast differentiation such as ID2 are activated whereas genes essential for myoblast differentiation and fusion such as MYOG and MYMK are repressed, and that in differentiating satellite cells, genes essential for myoblast fusion such as MYMK and muscle contraction such as MYH3 are activated whereas genes stimulating cell proliferation such as CCND1 and genes inhibiting myoblast differentiation such as ID2 are repressed (Fig. 8).

### Electronic supplementary material

Below is the link to the electronic supplementary material.

Supplementary Material 1

Supplementary Material 2

Supplementary Material 3

Supplementary Material 4

Supplementary Material 5

Supplementary Material 6

Supplementary Material 7

Supplementary Material 8

Supplementary Material 9

Supplementary Material 10

Supplementary Material 11

Supplementary Material 12

Supplementary Material 13

## Data Availability

The sequencing data from this study has been deposited in the NCBI GEO database (https://www.ncbi.nlm.nih.gov/geo/) under accession number GSE253395.

## Abbreviations

ABAM: Antibiotic-antimycotic
ChIP: Chromatin immunoprecipitation assay
ChIP-seq: Chromatin immunoprecipitation followed by sequencing
DMEM: Dulbecco’s Modified Eagle Medium
FBS: Fetal bovine serum
GO: Gene ontology
H3K27ac: Acetylation of lysine 27 of histone 3
H3K27me3: Tri-methylation of lysine 27 of histone 3
H3K4me1: Monomethylation of lysine 4 of histone 3
MEF2: Myocyte enhancer factor 2
MHC: Myosin heavy chain
MRF: Myogenic regulatory factor
MYOG: Myogenin
RT-qPCR: Reverse transcription-quantitative PCR
TSS: Transcription start site
