# Supplementary material for: Chromatin profiling reveals TFAP4 as a critical transcriptional regulator of bovine satellite cell differentiation
Source: BMC Genomics. 2024 Mar 12;25:272. doi: 10.1186/s12864-024-10189-2 (PMC10935830; doi:10.1186/s12864-024-10189-2)
Supplement: Supplementary file 1 — Supplementary Material 1 [file 12864_2024_10189_MOESM1_ESM.docx]

**Additional Table 1.** Sequences of PCR primers used in this study

| Gene  (Bos taurus) | Direction | Primer sequence | GenBank accession number |
| --- | --- | --- | --- |
| MYOG | Forward | 5’TGGGCGTGTAAGGTGTGTAA3’ | NM_001111325 |
|  | Reverse | 5’TATGGGAGCTGCATTCACTG3’ |  |
| MYH2 | Forward | 5’CTGGCTGGAGAAGAACAAGG3’ | NM_001166227.1 |
|  | Reverse | 5’CACCGTCTGGAAAGAAGAGC3’ |  |
| MYH3 | Forward | 5’CTGGAGGAAATGAGGGATGA3’ | NM_001101835 |
|  | Reverse | 5’CACTCTTGAGAAGGGGCTTG3’ |  |
| CKM | Forward | 5’TGGAGATGATCTGGACCCCA3’ | NM_174773.4 |
|  | Reverse | 5’TTTCCCCTTGAACTCACCCG3’ |  |
| MYMK | Forward | 5’GCTCGGCCATCCTCATCATT3’ | NM_001193046.1 |
|  | Reverse | 5’GTCCCAGTCCTCGAAGAAGAA3’ |  |
| TFAP4 | Forward | 5’TAGGAGGGCTCTGTAGCCTG3’ | NM_001101215.1 |
|  | Reverse | 5’CTTGAGGGACTGGAAGCCTG3’ |  |
| HMBS | Forward | 5’CTTTGGAGAGGAATGAAGTGG3’ | NM_001046207.1 |
|  | Reverse | 5’AATGGTGAAGCCAGGAGGAA3’ |  |
