# Supplementary material for: Chromatin profiling reveals TFAP4 as a critical transcriptional regulator of bovine satellite cell differentiation
Source: BMC Genomics. 2024 Mar 12;25:272. doi: 10.1186/s12864-024-10189-2 (PMC10935830; doi:10.1186/s12864-024-10189-2)
Supplement: Supplementary file 2 — Supplementary Material 2 [file 12864_2024_10189_MOESM2_ESM.docx]

**Additional Table 2.** Mapping summary of ChIP-seq and Input libraries

| Cells^1^ | Biol.  Rep.^2^ | Library | Total reads | Mapped reads | Mapping rate | Uniquely mapped reads | Unique mapping rate |
| --- | --- | --- | --- | --- | --- | --- | --- |
| pbsc | 1 | H3K4me1 | 18221627 | 16414877 | 90% | 13790254 | 84% |
| pbsc | 1 | H3K27me3 | 58297726 | 54654975 | 94% | 40676092 | 74% |
| pbsc | 1 | H3K27ac | 21448210 | 20299973 | 95% | 16840903 | 83% |
| pbsc | 1 | Input | 44938552 | 43755261 | 97% | 22828541 | 52% |
| dbsc | 1 | H3K4me1 | 45115302 | 43402794 | 96% | 35730444 | 82% |
| dbsc | 1 | H3K27me3 | 60622721 | 57940234 | 96% | 40612347 | 70% |
| dbsc | 1 | H3K27ac | 14353126 | 13734971 | 96% | 11247320 | 82% |
| dbsc | 1 | Input | 79090030 | 77004857 | 97% | 33174668 | 43% |
| pbsc | 2 | H3K4me1 | 62939262 | 56789377 | 90% | 51664518 | 91% |
| pbsc | 2 | H3K27me3 | 70412492 | 65924875 | 94% | 57013352 | 86% |
| pbsc | 2 | H3K27ac | 73126007 | 68331096 | 93% | 61047964 | 89% |
| pbsc | 2 | Input | 138034047 | 131450120 | 95% | 78138376 | 59% |
| dbsc | 2 | H3K4me1 | 61175600 | 58464487 | 96% | 51672028 | 88% |
| dbsc | 2 | H3K27me3 | 58971884 | 55939013 | 95% | 46729946 | 84% |
| dbsc | 2 | H3K27ac | 48967804 | 46113867 | 94% | 40306384 | 87% |
| dbsc | 2 | Input | 152747059 | 144905539 | 95% | 69369284 | 48% |

^1^ pbsc, proliferating bovine satellite cells; dbsc, differentiating bovine satellite cells. ^2^ biological replicate.
