# Supplementary material for: Chromatin profiling reveals TFAP4 as a critical transcriptional regulator of bovine satellite cell differentiation
Source: BMC Genomics. 2024 Mar 12;25:272. doi: 10.1186/s12864-024-10189-2 (PMC10935830; doi:10.1186/s12864-024-10189-2)
Supplement: Supplementary file 3 — Supplementary Material 3 [file 12864_2024_10189_MOESM3_ESM.docx]

**Additional Table 3.** Quality assessment of ChIP-seq libraries

| Cells | Biol. Rep. | Library | NSC | RSC | Qtag^1^ |
| --- | --- | --- | --- | --- | --- |
| pbsc | 1 | H3K4me1 | 1.04245 | 1.09585 | 1 |
| pbsc | 1 | H3K27me3 | 1.01305 | 0.88422 | 0 |
| pbsc | 1 | H3K27ac | 1.12573 | 1.06880 | 1 |
| dbsc | 1 | H3K4me1 | 1.04105 | 1.08982 | 1 |
| dbsc | 1 | H3K27me3 | 1.01304 | 0.74078 | 0 |
| dbsc | 1 | H3K27ac | 1.12339 | 1.04489 | 1 |
| pbsc | 2 | H3K4me1 | 1.06609 | 1.89992 | 2 |
| pbsc | 2 | H3K27me3 | 1.03774 | 2.77064 | 2 |
| pbsc | 2 | H3K27ac | 1.12925 | 1.3082 | 1 |
| dbsc | 2 | H3K4me1 | 1.06756 | 2.01047 | 2 |
| dbsc | 2 | H3K27me3 | 1.04587 | 3.08938 | 2 |
| dbsc | 2 | H3K27ac | 1.1443 | 1.30041 | 1 |

^1^Qtag (quality tag) is based on RSC, where −2 means very low quality, −1 low quality, 0 medium quality, 1 high quality, and 2 very high quality.
