# Supplementary material for: Chromatin profiling reveals TFAP4 as a critical transcriptional regulator of bovine satellite cell differentiation
Source: BMC Genomics. 2024 Mar 12;25:272. doi: 10.1186/s12864-024-10189-2 (PMC10935830; doi:10.1186/s12864-024-10189-2)
Supplement: Supplementary file 13 — Supplementary Material 13 [file 12864_2024_10189_MOESM13_ESM.pdf]

Original images for Fig.6 (TFAP4 knockdown experiment)

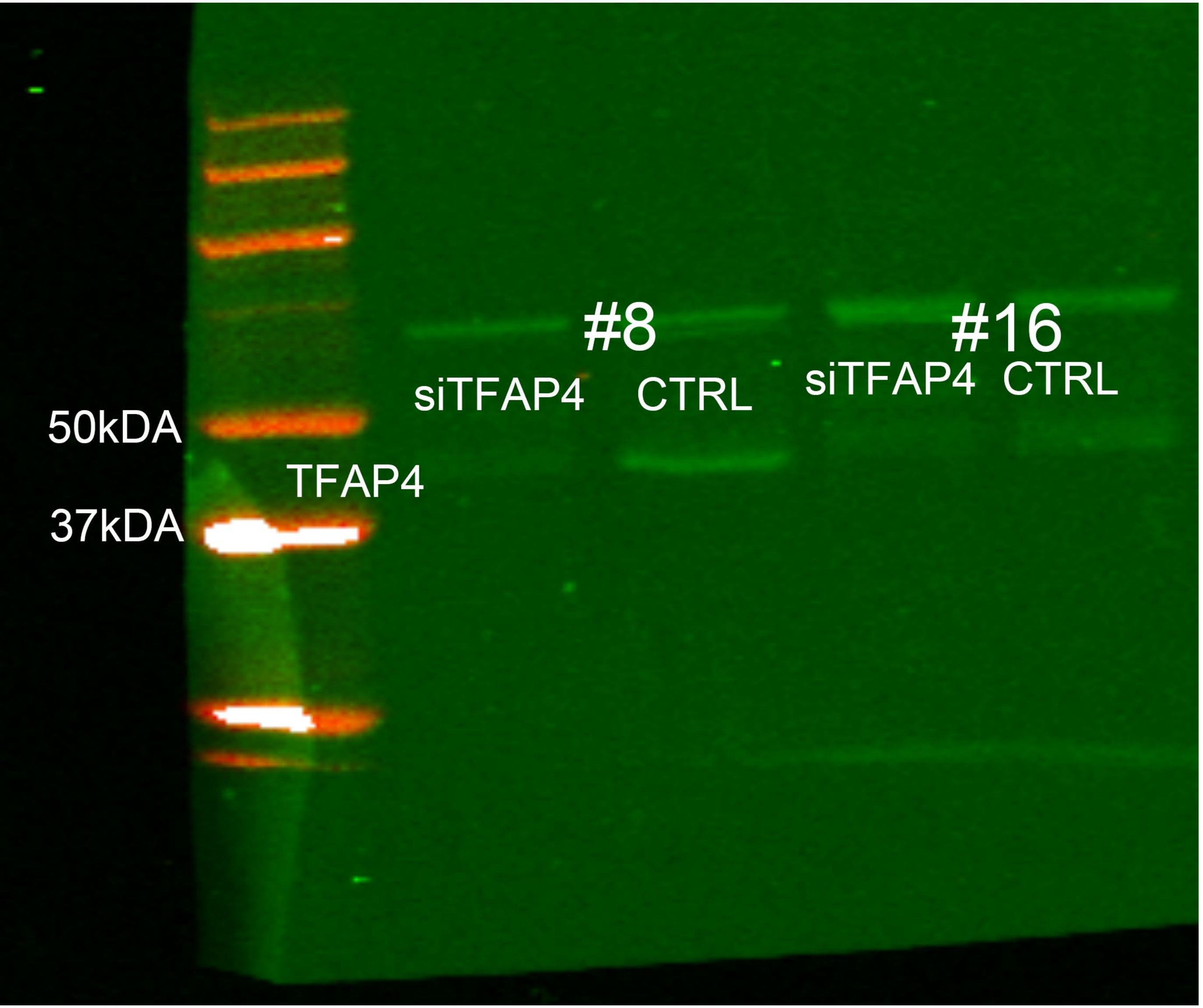

The image shown in the manuscript was from sample #8.

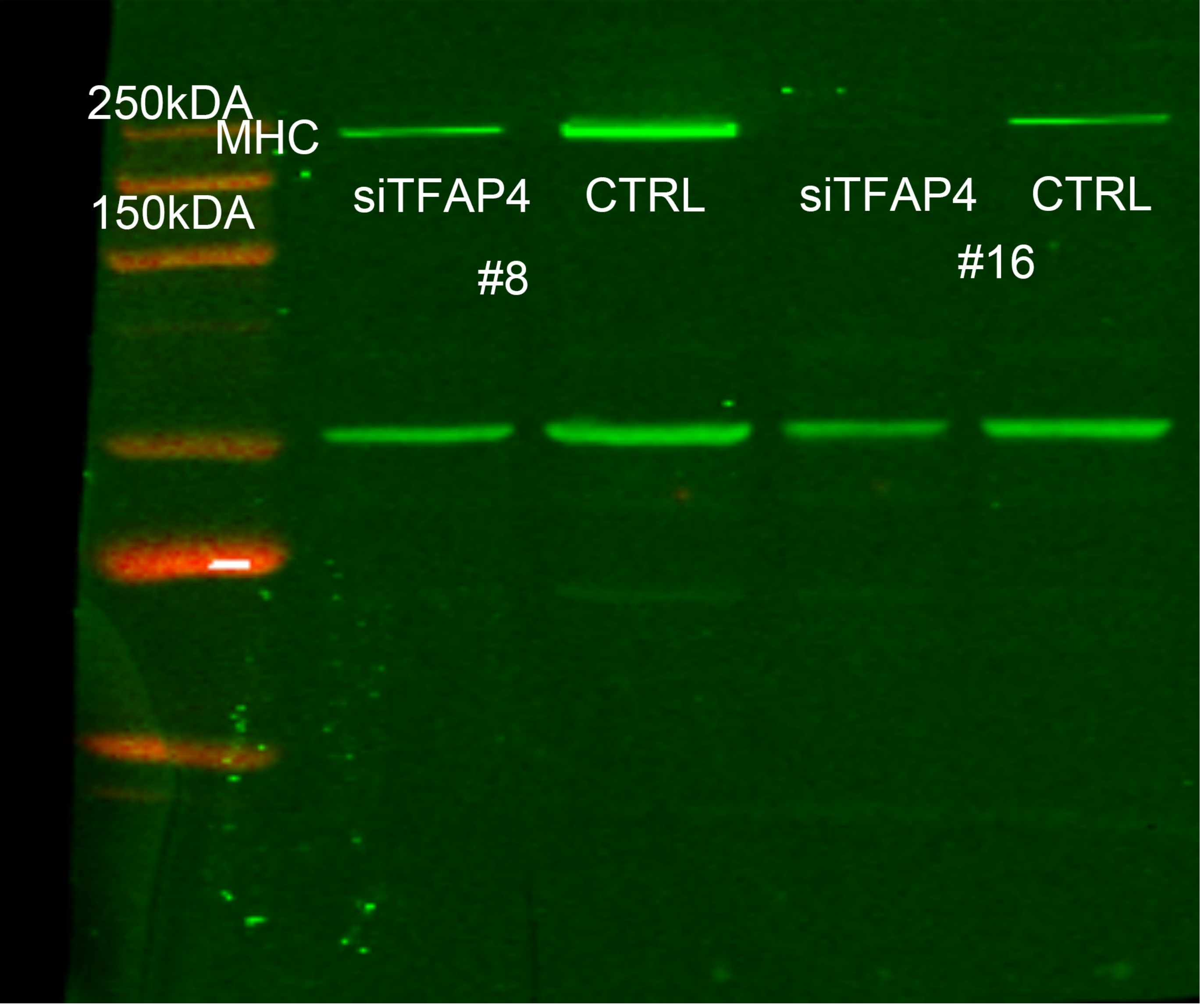
250kDA
MHC
150kDA
siTFAP4
CTRL
siTFAP4
CTRL
#8
#16

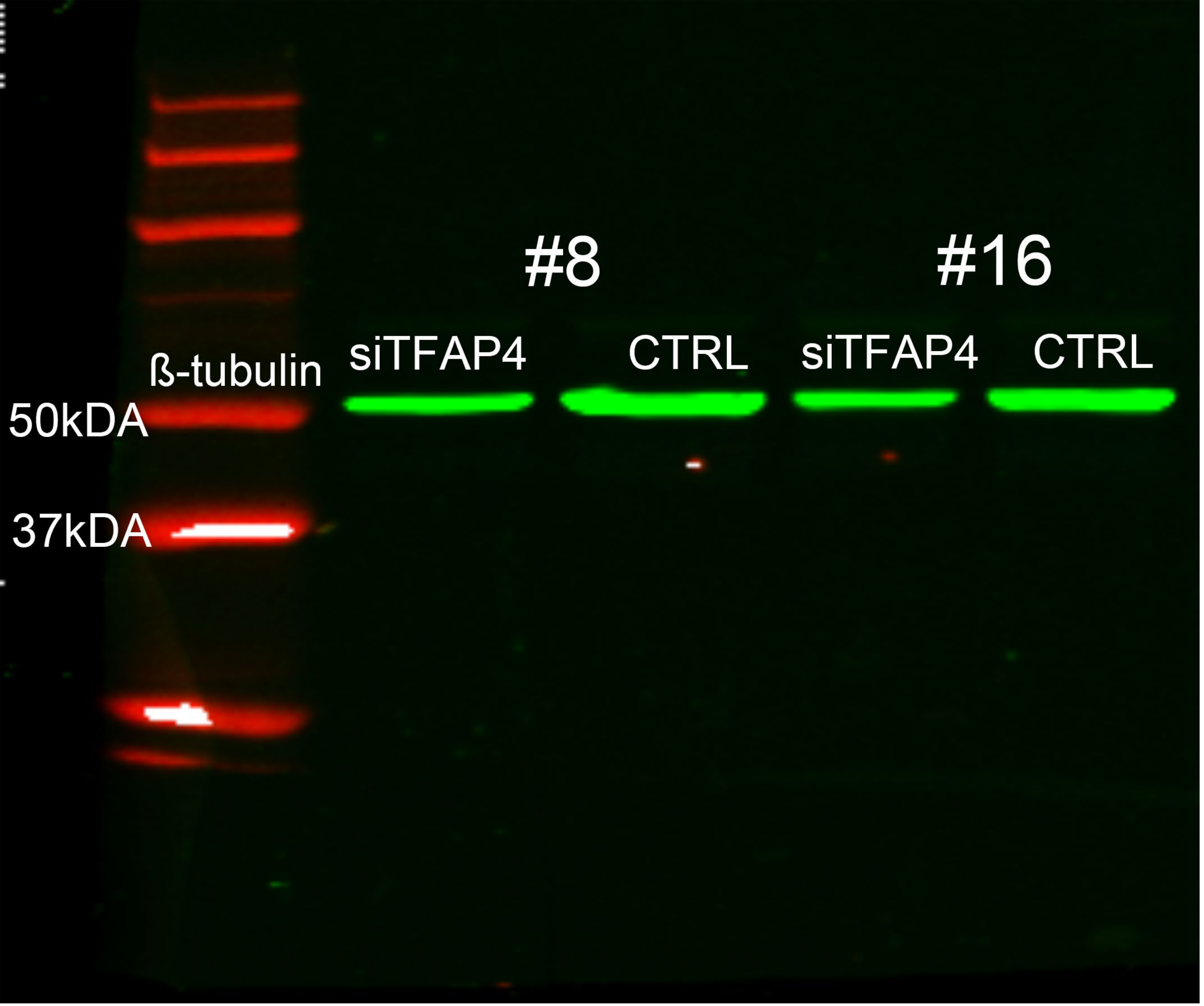
#8
#16
ß-tubulin
siTFAP4
CTRL
siTFAP4
CTRL
50kDA
37kDA

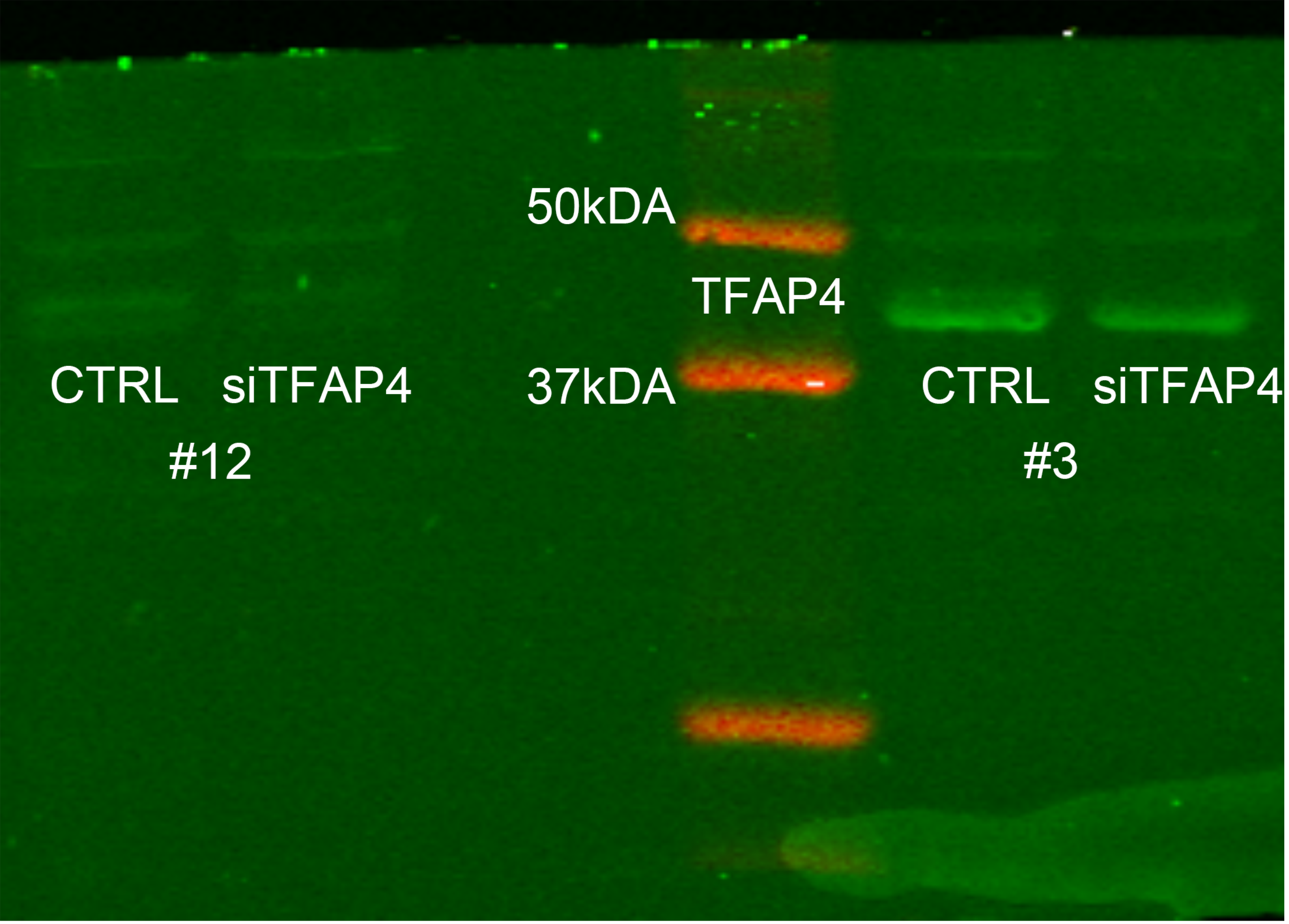
50kDA
TFAP4
CTRL
siTFAP4
37kDA
CTRL
siTFAP4
#12
#3

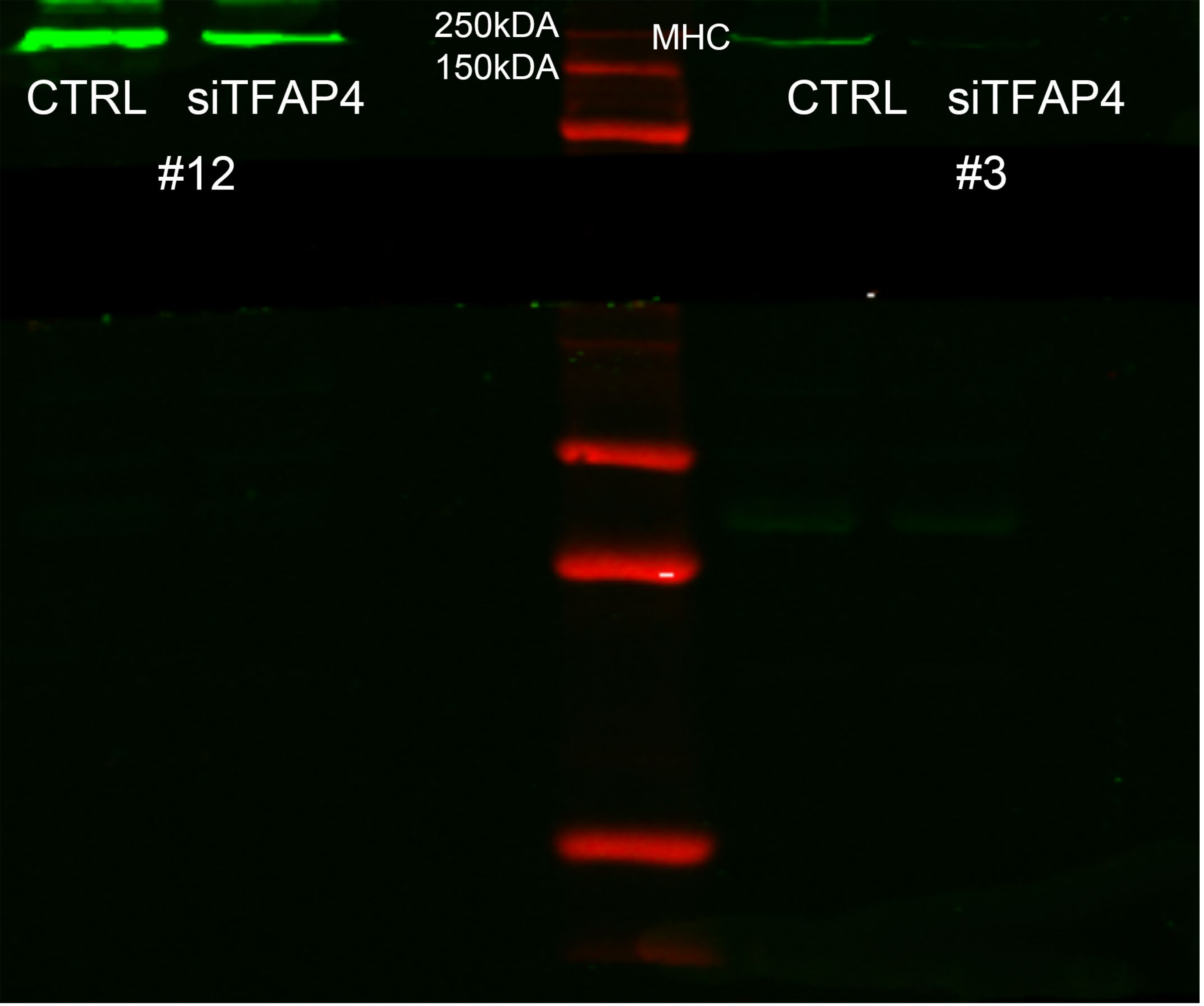
250kDA
150kDA
MHC
CTRL
siTFAP4
#12
CTRL
siTFAP4
#3

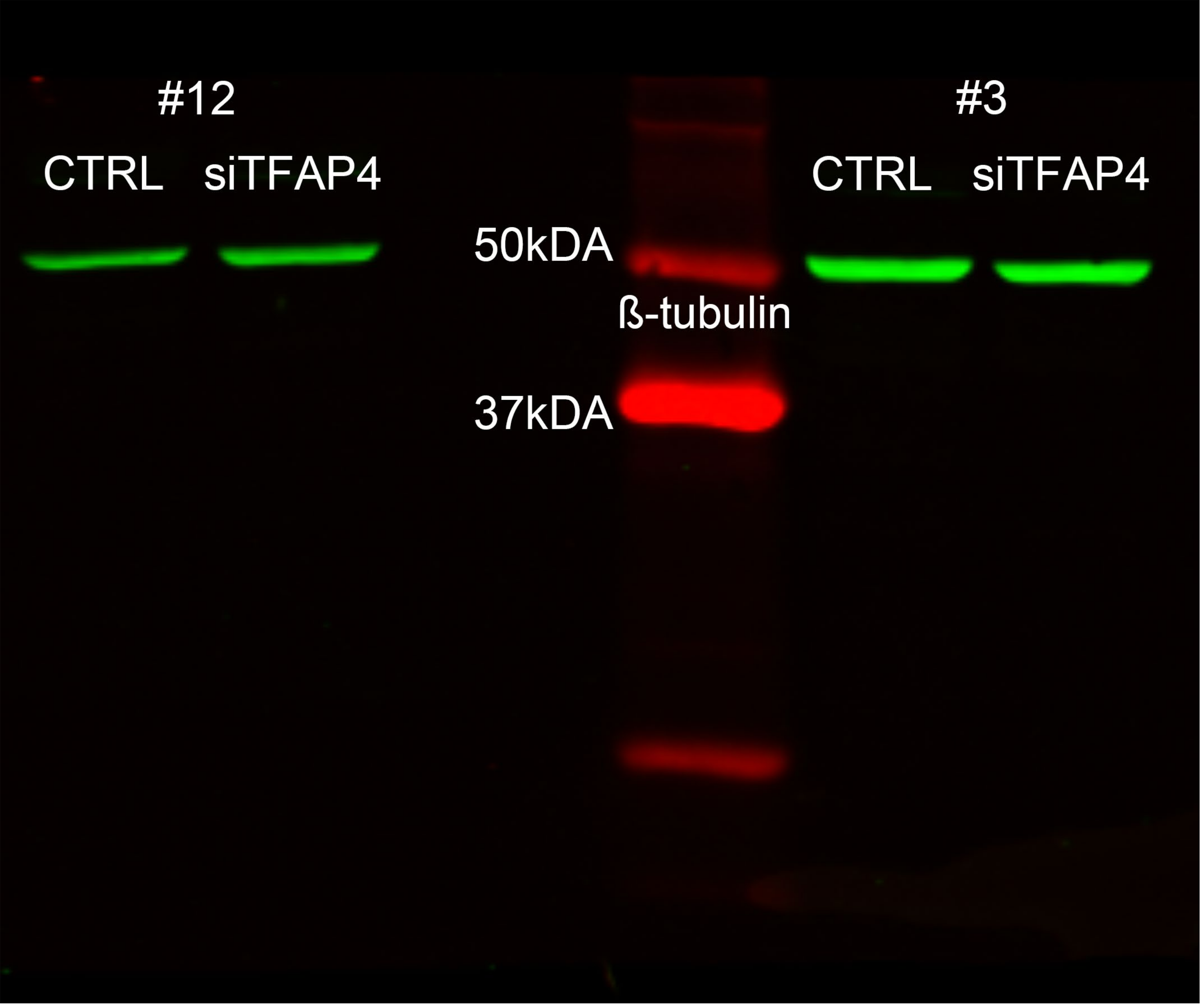
#12
CTRL
siTFAP4
#3
CTRL
siTFAP4
50kDA
ß-tubulin
37kDA

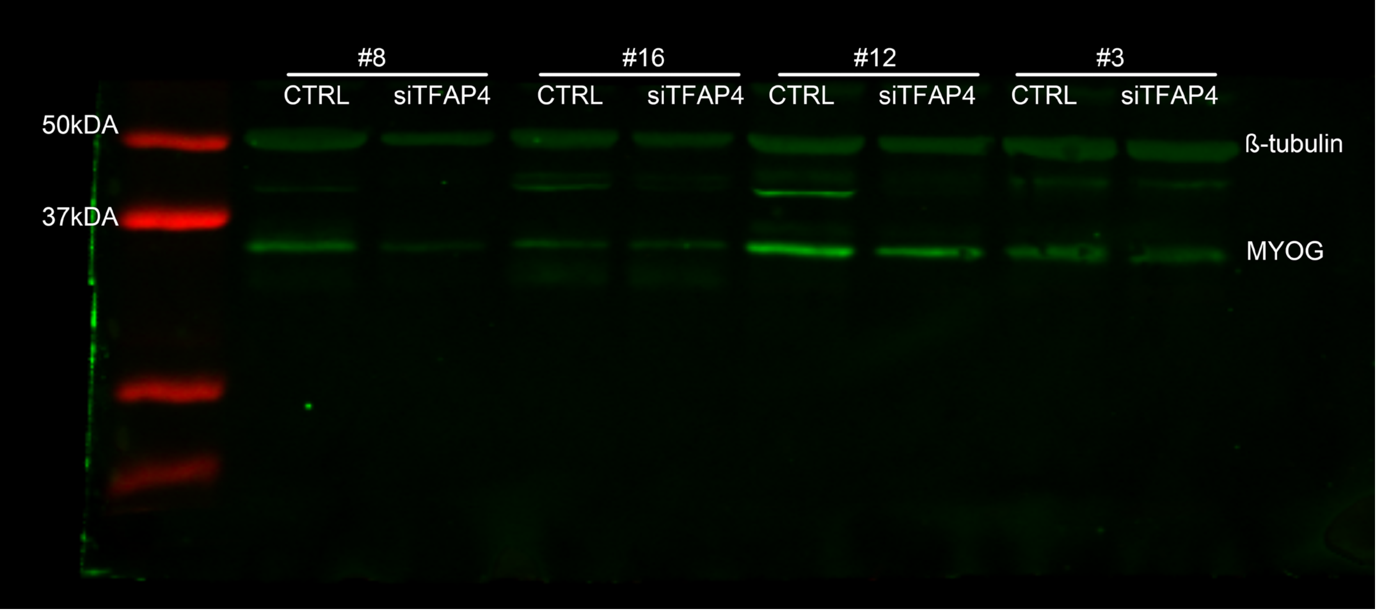
#8
#16
#12
#3
CTRL
siTFAP4
CTRL
siTFAP4
CTRL
siTFAP4
CTRL
siTFAP4
50kDA
37kDA
ß-tubulin
MYOG

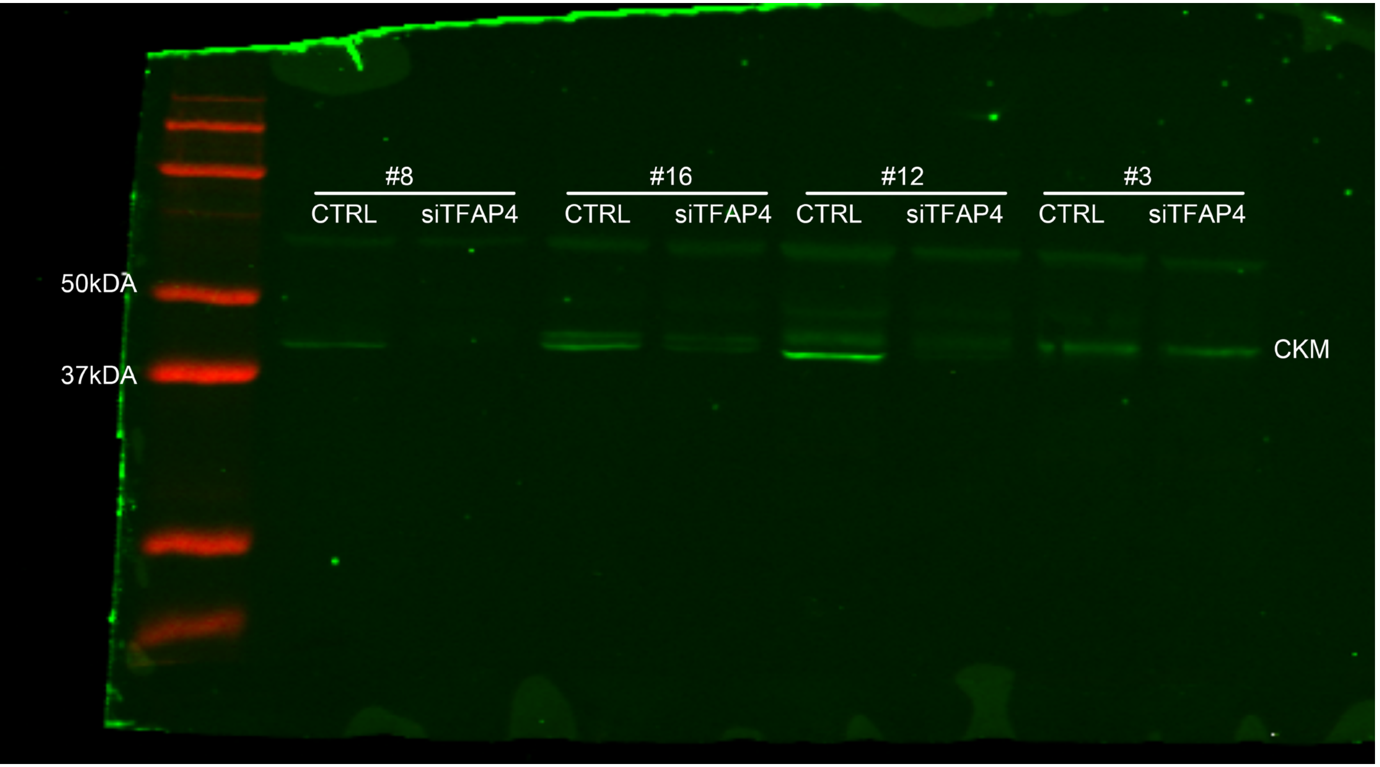
#8
#16
#12
#3
CTRL
siTFAP4
CTRL
siTFAP4
CTRL
siTFAP4
CTRL
siTFAP4
50kDA
37kDA
CKM

Original images for Fig.7 (TFAP4 overexpression experiment)

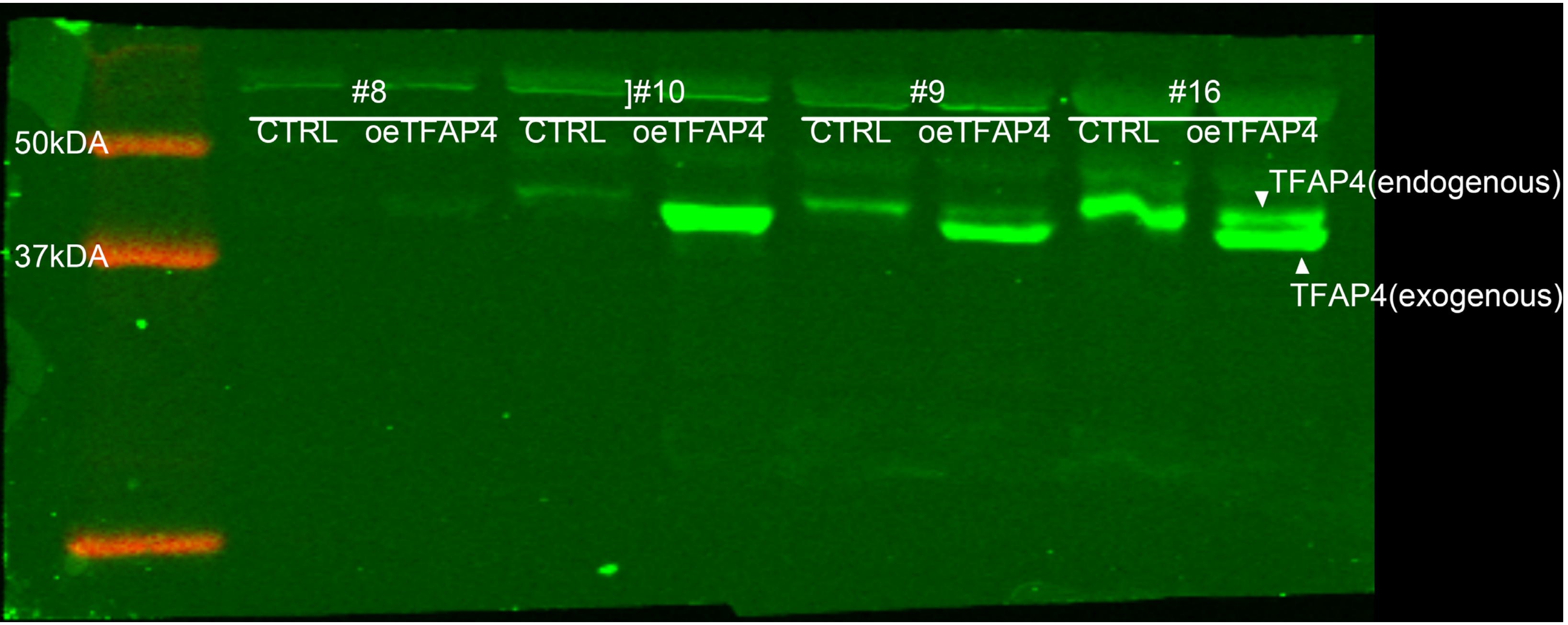

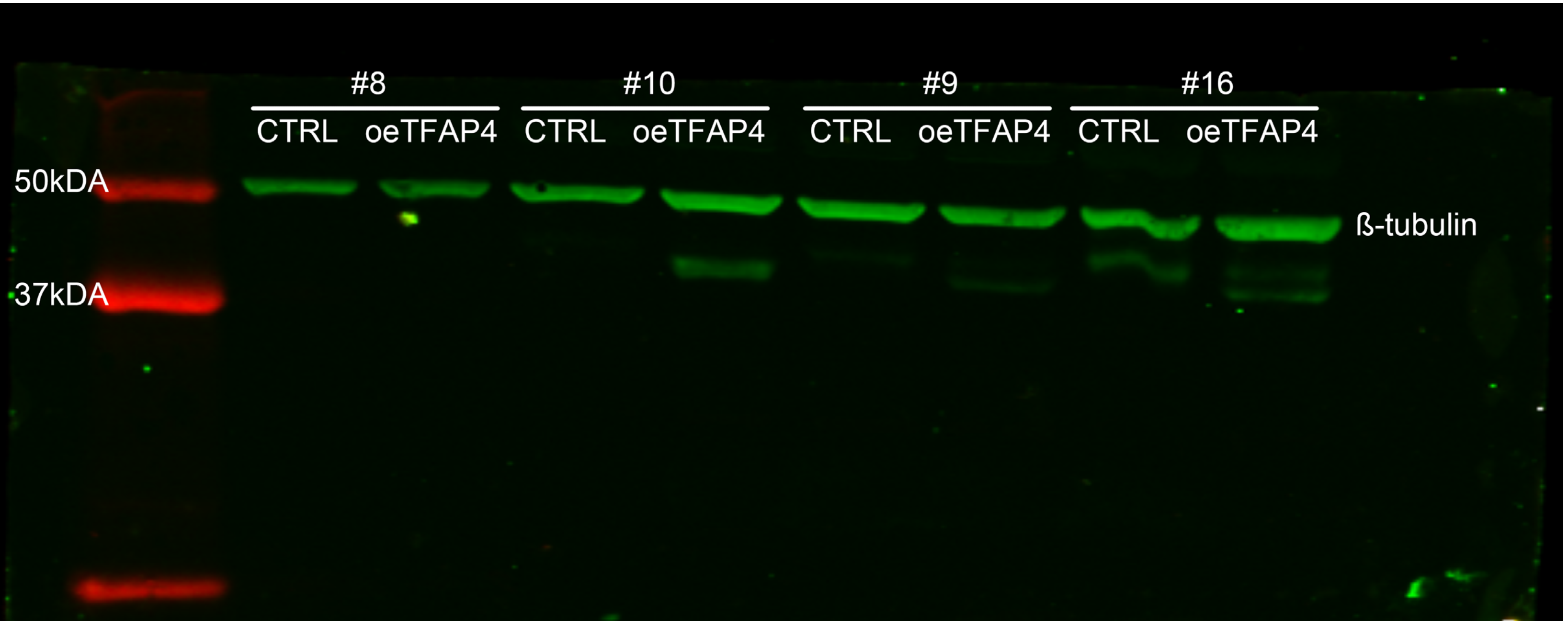

There are two gels shown in the manuscript.

One gel contains TFAP4 from sample #10, and the other gel contains MHC, CKM, MYOG, and ß-tubulin, from sample #9. To save space, ß-tubulin from #10 is not shown.

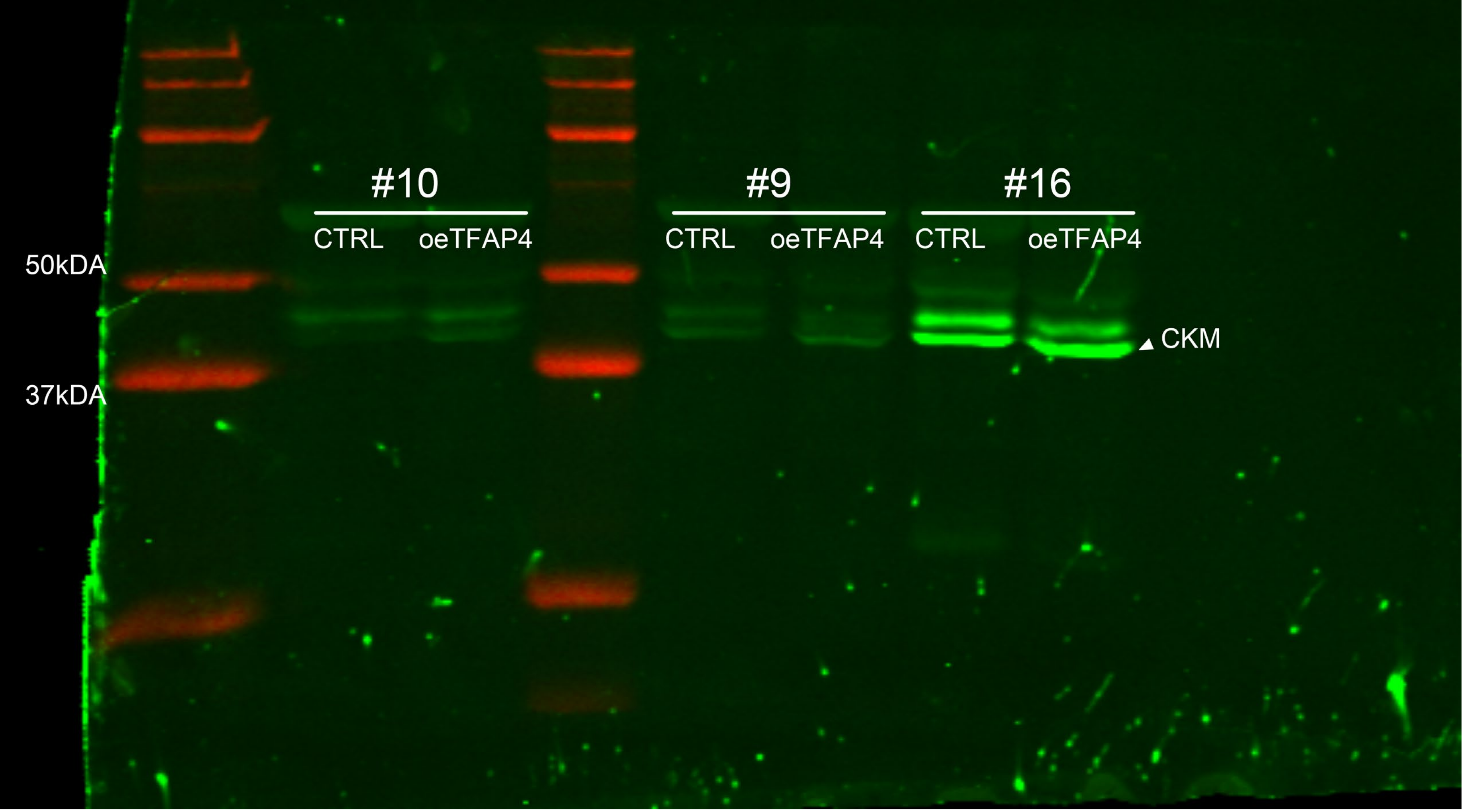

#10
#9
#16
CTRL
oeTFAP4
CTRL
oeTFAP4
CTRL
oeTFAP4
50kDA
37kDA
CKM

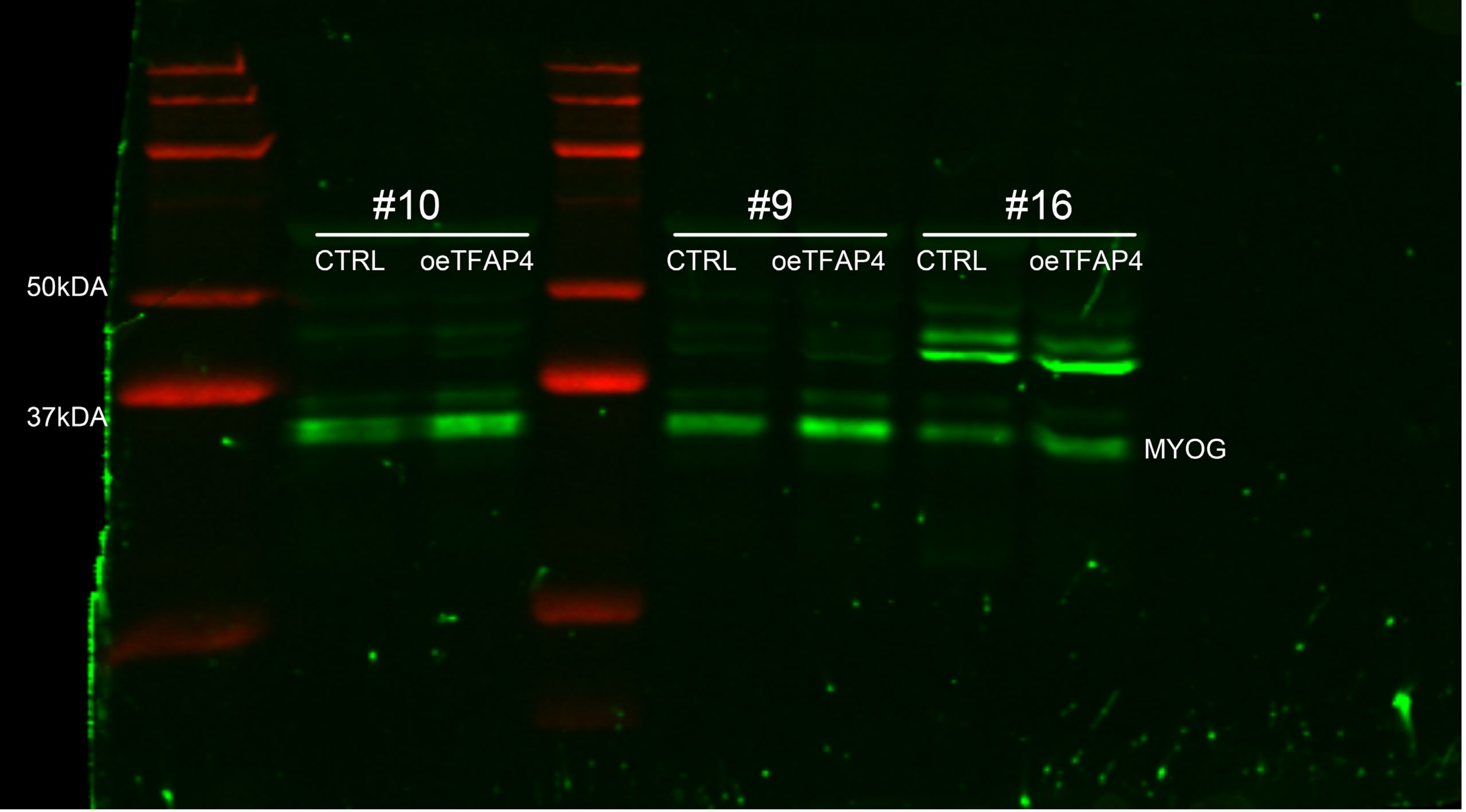
50kDA
37kDA
#10
CTRL
oeTFAP4
#9
CTRL
oeTFAP4
#16
CTRL
oeTFAP4
MYOG

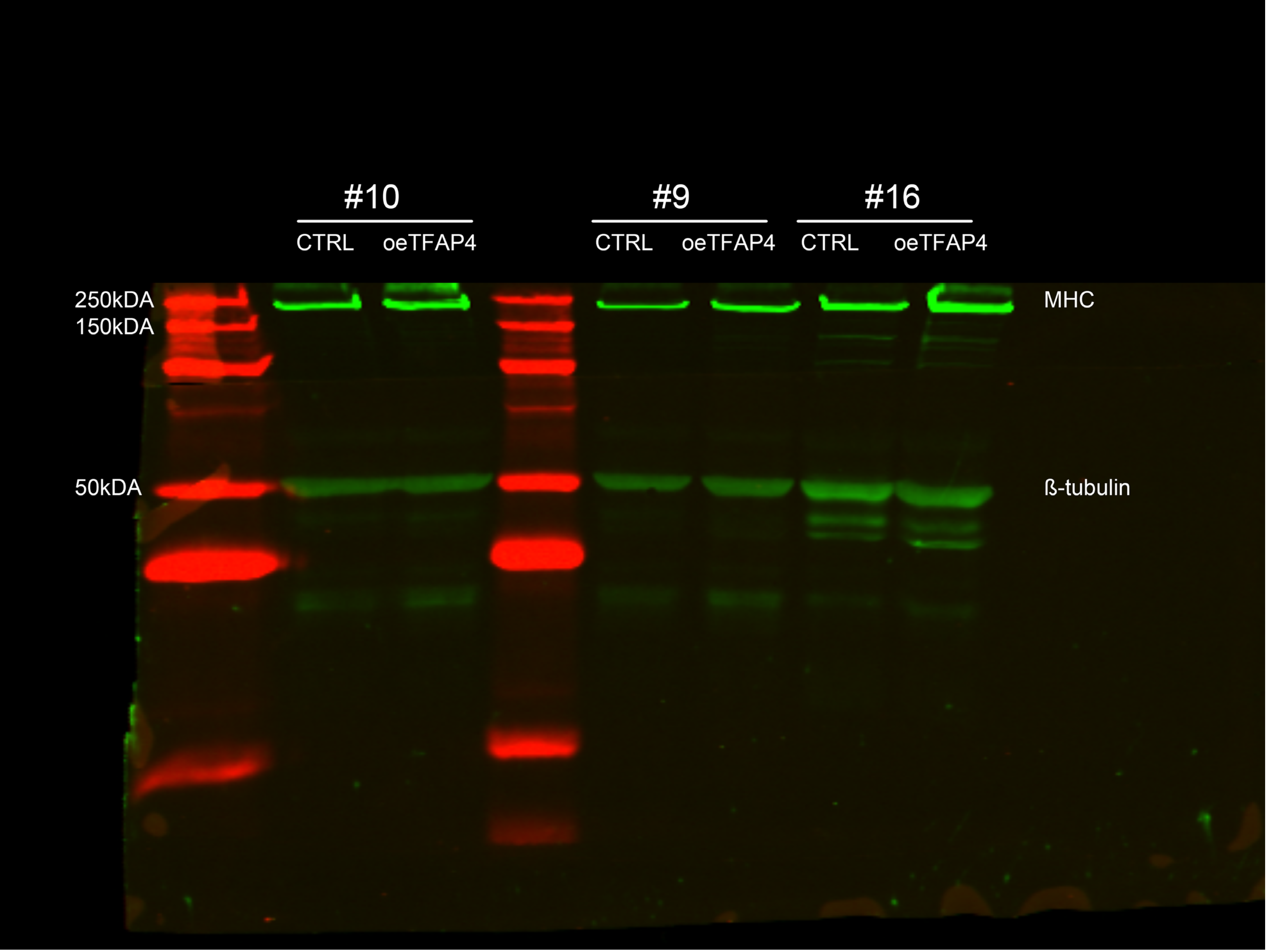
#10
#9
#16
CTRL
oeTFAP4
CTRL
oeTFAP4
CTRL
oeTFAP4
250kDA
150kDA
50kDA
MHC
ß-tubulin
